# Nanofiber Networks from Self-Assembling Cardanol Amphiphiles: Toward Renewable Multifunctional Surfactants

**DOI:** 10.3390/molecules30204119

**Published:** 2025-10-17

**Authors:** Yichuan Wang, Leilei Zhao, Bao Liu, Longhui Deng, Zhenqiang Wu

**Affiliations:** 1 School of Biology and Biological Engineering, South China University of Technology, Guangzhou 510006, China; wangyichuan@lonkey.com.cn; 2 Lonkey Industrial Co., Ltd., Guangzhou 511400, China; 3 Hongmian Zhihui Science and Technology Innovation Co., Ltd., Guangzhou 510630, China

**Keywords:** multifunctional surfactant, cardanol, self-assemble, renewable resources

## Abstract

This article focuses on the utilization of the supramolecular self-assembly of renewable materials derivatives to obtain functional compounds. Novel bio-based amphiphile molecules (**CALAH** and **PALAH**) were synthesized through a tailored process, involving Williamson ether synthesis and amidation reactions, employing renewable amino acid and cashew nut shell liquid (CNSL) derivatives as essential reactants. Their molecular structures were confirmed by nuclear magnetic resonance (NMR), high-resolution mass spectrometry (HRMS), and Fourier-transform infrared spectroscopy (FT-IR). Notably, these compounds self-assemble into nanofibers that organize into a fibrous network, unexpectedly exhibiting two distinct morphologies: curved and rigid nanostructures. These structures were characterized by scanning electron microscopy (SEM), and their formation mechanisms were elucidated through temperature-dependent NMR studies and density functional theory (DFT) calculations. The sodium salts of the compounds (**PALA** and **CALA**) exhibited fundamental surfactant properties, exhibiting a hydrophilic lipophilic balance (HLB) value of 13.7 and critical micelle concentration (CMC) values of 1.05 × 10^−5^ M and 4.10 × 10^−6^ M. They also demonstrated low cytotoxicity, suggesting potential suitability in consumer applications. Furthermore, the compounds exhibited multi-functional performance as effective inhibitors of *Staphylococcus aureus* and efficient adsorbents for gaseous pollutants.

## 1. Introduction

As societal development advances, resource and environmental challenges have become increasingly prominent and can no longer be overlooked [1,2,3,4]. Meanwhile, surfactants represent essential raw materials in modern industrial systems. In this context, bio-based surfactants demonstrate considerable advantages over their petroleum-based counterparts [5,6]. They are sourced from abundant and renewable raw materials that can be sustainably harvested on a seasonal basis [7,8]. Moreover, they exhibit inherent biodegradability, with degradation times significantly shorter than those of petroleum-derived alternatives [9,10,11]. A number of bio-based surfactants have already been successfully commercialized, such as alkyl polyglycolides (APGs) [12,13,14] derived from sugars [15], lignin [16,17], and glycerides [18,19,20].

Over the past two decades, cashew nut shell liquid (CNSL), a natural extract obtained from cashew nutshell biomass, has attracted substantial research interest as a promising renewable feedstock [21,22,23]. It has been employed in the synthesis of surfactants for the textile industry and as a substitute for phenolic resins [24]. More recently, CNSL has also been explored as a precursor for pharmaceutical compounds [25,26]. Upon heating and decarboxylation, this biomass yields cardanol, which features a natural hydrophobic pentadecyl phenolic chain [27].

**Scheme 1:**
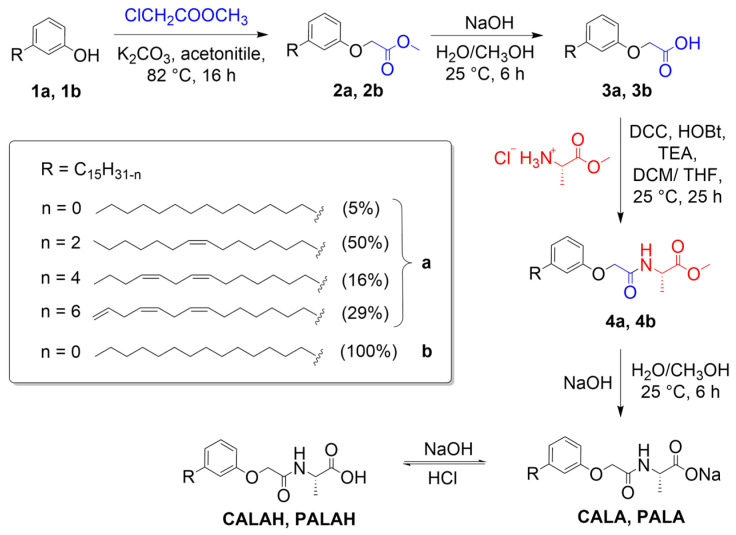
Synthetic route of amphiphilic molecules **CALAH** and **PALAH**.

In this work, we report for the first time the synthesis of novel bio-based amphiphilic molecules using CNSL and alanine as the starting materials. Amino acids represent a diverse and abundant resource; herein, an L-alanine derivative was selected as the hydrophilic precursor [28,29]. Unlike synthetically produced D-alanine, which can exhibit a level of toxicity, L-alanine is a naturally occurring non-essential amino acid that is well-tolerated in the human body [30]. It serves as the hydrophilic segment of the amphiphile, terminating in a carboxylate or sodium carboxylate group. The hydrophobic moiety was constructed from two processed derivatives of CNSL: one consisting of a mixture of cardanol (**1a**), and the other its hydrogenated analog, 3-pentadecylphenol (**1b**). Cardanol comprises a mixture of unsaturated hydrocarbons with four levels of saturation (Ω = 0, 1, 2, and 3), all containing non-conjugated double bonds (see Scheme 1) [25,31].

Our experiments yielded the unexpected discovery that the final amphiphilic molecules synthesized from two distinct structural raw materials exhibited significantly divergent properties, which contrasts with the surfactant molecules previously synthesized using CNSL as the raw material. Since the structure of this molecule is pentadecylphenoxy acetyl-*L*-alanine, and its hydrophilic portion is capable of forming intermolecular hydrogen bonds with other identical molecules in both lateral and longitudinal directions, this portion can self-assemble into tubular structures and aggregate into a network configuration. A thorough investigation was conducted to elucidate the underlying mechanisms responsible for this observation, and the property was employed to assess its surfactant behavior. Additionally, toxicity and gas pollutant adsorption tests showed low toxicity and multifunctional adsorption characteristics, suggesting potential application value in the personal care and cosmetics industries.

## 2. Results and Discussion

### 2.1. Synthesis of Amphiphilic Molecules **CALAH** and **PALAH**

The synthetic route for these molecules is illustrated in Scheme 1. To obtain the amide bond connected to the amino acid, we first used methyl chloroacetate and phenol (**1a** and **1b**) as raw materials to perform the Williamson ether synthesis reaction, followed by ester hydrolysis to obtain pentadecylphenoxyacetic acid (**3a** and **3b**). Subsequently, the neutralized L-alanine methyl ester hydrochloride by triethylamine (TEA) was reacted with the activated carboxylic acid product **3a** and **3b** by *N*,*N*′-dicyclohexylcarbodiimide (DCC) and 1-hydroxybenzotriazole (HOBt) to perform an amidation reaction, followed by ester hydrolysis to obtain the target products **CALA**, sodium (cardenolphenoxy)acetyl-*L*-alanine, and **PALA**, sodium (3-pentadecylphenoxy)acetyl-*L*-alanine. After acidification, the carboxylic acid products were **CALAH**, (cardenolphenoxy)acetyl-*L*-alanine, and **PALAH**, (3-pentadecylphenoxy)acetyl-*L*-alanine. The intermediates of each step were characterized by NMR and HRMS. Additionally, the two final products were analyzed via FT-IR (see Supplementary Materials, Figures S1–S20, which show the spectra of all of the compounds).

According to the HRMS spectrum, corresponding fragment signals can be found for both target molecules, while **CALAH** exhibits distinct fragment signals with *n* values of 0, 2, 4, and 6. The FT-IR spectrum indicates that the presence of an additional double bond in **CALAH** leads to the generation of more complex signals in the fingerprint region when compared to **PALAH**. Furthermore, an elongated alkyl chain contributes to the diminution of the broad peak of the carboxyl group, which can be verified in the NMR spectrum with deuterated DMSO as the solvent. Additionally, the appearance of signals at 3390 cm^−1^ and 3320 cm^−1^ suggests that the spatial configuration of the NH group within the amide bond is restricted due to the introduction of chiral L-alanine. Conversely, **PALAH** only has one strong signal, which suggests that these two molecules manifest distinct microscopic structural features.

**Figure 1:**
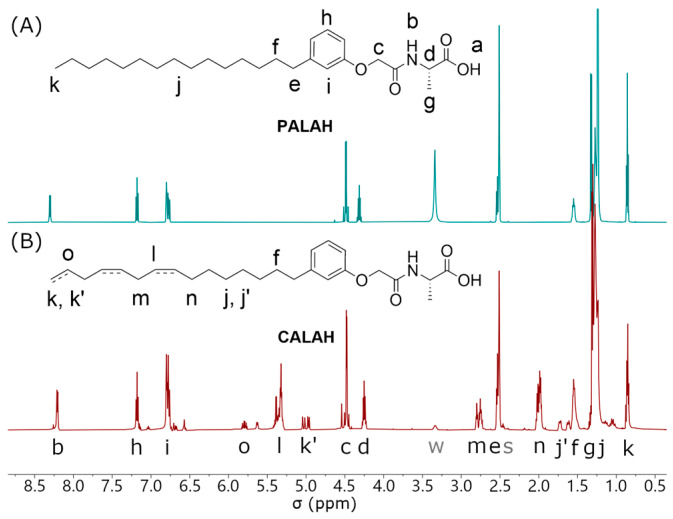
^1^H NMR spectra (600 MHz, DMSO-*d*_6_, ppm, 25 °C) of (**A**) **PALAH** and (**B**) **CALAH**. The two resonances centered at 3.33 ppm (gray “w”) and 2.51 ppm (gray “s”) are attributed to the protons of the solvents DMSO and water, respectively.

The structural content ratios of the four distinct carbon chains present in the raw material cardanol are indicative of the quantities of the final products synthesized, which also conform to this ratio. Figure 1 presents a comparison of the ^1^H NMR spectra of **CALAH** and **PALAH** (600 MHz, DMSO-*d*_6_, ppm, 25 °C). For **PALAH**, the proton assignments are unambiguous. Meanwhile, the augmented proton resonance signals of **CALAH**, in contrast to **PALAH**, are all attributed to the double bonds in the extended alkene chain. It was determined that three of these double bonds account for 29% of the structure. Therefore, the protons *k*′ and *o* from the terminal alkene exhibit resonance signal integrals of approximately one-third of a single proton, with shifts at 5.00 ppm and 5.80 ppm, respectively. Meanwhile, the resonance signal integral of the corresponding proton *k* (0.85 ppm) is 2, which exactly corresponds to two-thirds of the alkyl integral. The alkyl proton *m* between the two isolated double bonds has a resonance signal integral of 1.5, which also corresponds to four protons in the structure with three double bonds (29%) and two protons in the structure with two double bonds (16%). The remaining two distinct resonance signals are the proton *b* on the amide bond N, the proton *g* on the adjacent methyl group of the amide, and the protons *j* (*j*′) on the remaining alkyl carbon chain. This observed difference is attributable to the proton *j* on the alkyl carbon chain of **CALAH** shifting downfield in comparison to the proton resonance signal of **PALAH**, while *b* and *g* shift upfield. Thus, the underlying causes of this phenomenon vary. The amalgamation of these two amphiphilic molecules results in distinct final forms, with **PALAH** manifesting as a powdery solid and **CALAH** assuming a viscous solid state. This observation aligns with the disparate macroscopic states and properties exhibited by these molecules.

### 2.2. Morphological Structures

A morphological characterization of **CALAH** and **PALAH** was performed, with the basis of this characterization being the different structures of the two amphiphilic molecules.

**Figure 2:**
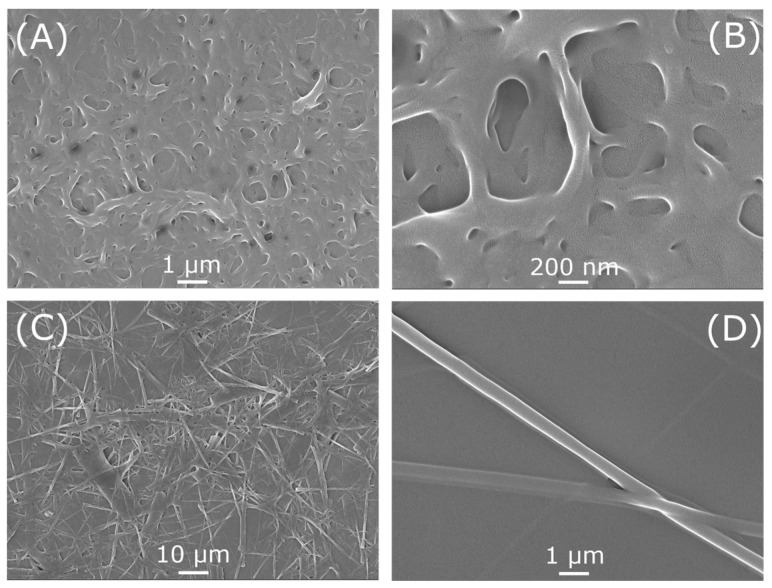
SEM images of **PALAH** (**A**,**B**) and **CALAH** (**C**,**D**), respectively.

Figure 2 presents a series of detailed images obtained through SEM. In the sample prepared by dispersing the mixture of water and ethanol, the two molecules surprisingly exhibited different fiber network structures. The morphology of **PALAH** at 1 μm (A) and 200 nm (B) is depicted as a network of soft curved tubular structures, while the morphology of **CALAH** at 10 μm (C) and 1 μm (D) is shown as a network of straight tubular structures. The diameters of these two species differ slightly, with **PALAH** exhibiting a smaller diameter compared to **CALAH**, and the diameters are not uniform. It can be reasonably posited that the structural differences observed in the carboxyl groups at the ends of the amphiphilic molecules result in the formation of dimers through O-H···O hydrogen bonds. These dimers subsequently aggregate through N-H···O hydrogen bonds via the amide groups, thereby leading to the formation of tubular structures.

Two other aprotic solvents, ethyl acetate and cyclohexane, were used as solvents for SEM analysis, which is shown in Figures S25 and S26 of the Supplementary Materials. It can be seen that in ethyl acetate, **PALAH** forms the structures that common organic surfactants should form, such as needles, a film, and a block. Most of the **CALAH** is in an amorphous state, with only a few micellar vesicles. However, the difference is that in cyclohexane with very low polarity, fibers also only appeared in **CALAH**, but their lengths are shorter.

To corroborate this result, corresponding comparative tests were conducted using the carboxylate forms of the two molecules (**CALA** and **PALA**). As illustrated in Figure S24, the two sodium salt molecules form vesicles of varying sizes, akin to conventional surfactants. However, they aggregate randomly, without forming tubular structures or networks.

Furthermore, the optical rotation test proved that the structure of *L*-alanine did not undergo racemization after several synthesis processes, and the final c product remained chiral. The specific rotation of **CACAH** ${\left[\alpha \right]}_{D}^{20}$ = +292.461 (*c* 0.025, EtOH). The specific rotation of **PACAH** ${\left[\alpha \right]}_{D}^{20}$ = +293.395 (*c* 0.025, EtOH).

### 2.3. ^1^H NMR Self-Assembled Structures

**Figure 3:**
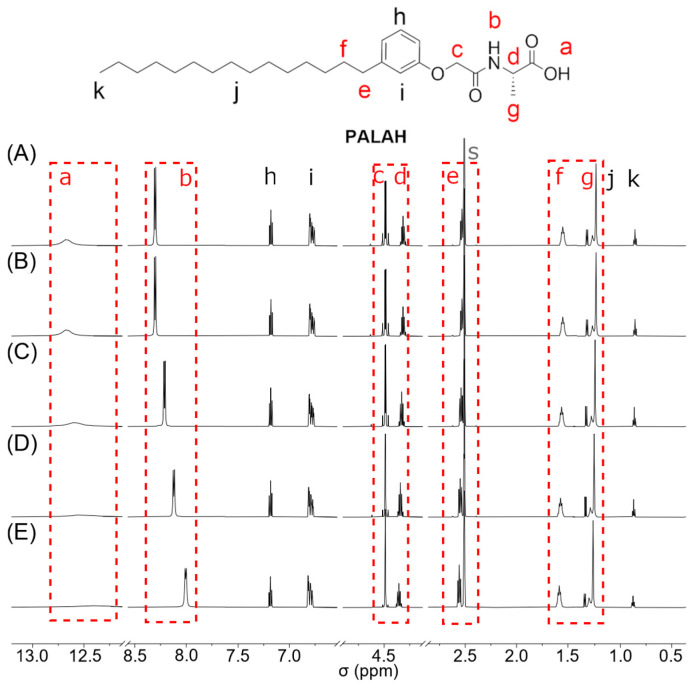
^1^H NMR spectra of temperature-dependent (600 MHz, DMSO-*d*_6_, ppm) for **PALAH**, (**A**) 10 °C, (**B**) 25 °C, (**C**) 40 °C, (**D**) 55 °C, (**E**) 70 °C.

We performed ^1^H NMR analysis of the self-assembly behavior of the two molecules, testing them under variable temperature NMR conditions. Figure 3 illustrates the hydrogen spectrum of **PALAH** as a function of temperature, with 15 °C intervals ranging from 10 °C to 70 °C. The results indicate that the most significant change occurs in the proton on the carboxylic acid, which gradually shifts to higher fields and disappears as the temperature increases. Conversely, the proton on the amide group also shifts upfield, resulting in a decrease in signal. Consequently, it can be deduced that both the carboxylic acid and the amide form intermolecular hydrogen bonds, manifesting a substantial de-shielding effect. As the temperature rises, the hydrogen bonds gradually weaken. The signal at 4.48 ppm demonstrates a *dd* pattern at low temperatures, resulting from the coupling splitting of protons on the amide group. As the temperature increases, the effect gradually diminishes, leading to a single signal. It is noteworthy that certain protons, specifically those located in proximity to the benzene ring (*e*, *f*) and the hydrogen bond (*g*), exhibited a shift in field strength to lower values ranging from 0.03 to 0.05 ppm. Concurrently, protons positioned along the alkyl chain also underwent a shift in field strength to lower values, ranging from 0.01 to 0.02 ppm. Proton shifts are known to typically not exceed 0.01 ppm; however, **PALAH** displays a distinct pattern of behavior. This finding also provides indirect evidence that, in the self-assembled state, the distances between the benzene rings and alkyl chains of the molecules are minimal, enabling interaction via van der Waals forces. As the temperature increases, the energy levels also rise, thereby strengthening the intermolecular de-shielding effect. This, in turn, causes a downfield shift in the protons.

### 2.4. DFT Calculations

To further demonstrate that amphiphilic molecules form two types of hydrogen bonds, O-H··· and N-H···O, and that intermolecular van der Waals interactions, including π–π interactions, exist between molecules, we performed DFT calculations on the two structures [32]. The calculations yielded the energy-minimized structures of the two molecules, as well as the dimer formed by the carboxylic acid and the tetramer formed by the two hydrogen bonds. As demonstrated in Figure S23, the separation between the O and H atoms of the carboxylic acid in the two molecules is 1.64 Å, which approaches the distance of a covalent bond. Meanwhile, the separation between the O and H atoms of the amide is 2.16 Å. Furthermore, the distance between the two benzene rings is 3.56 Å, and the distance between the H atoms of the alkyl chains ranges from 2.5 to 2.9 Å.

Preliminary energy calculations indicate that the binding energy of the tetramer is more than double that of the dimer, as shown in Table 1 [33]. This observation aligns with the findings from the preceding NMR results, which indicated that the binding energy of the dimer is predominantly attributed to hydrogen bonds. In contrast, the tetramer also incorporates van der Waals interactions from the terminal alkane chains. The binding energies of the terminal alkane chains in the two structures are also different. The carbon–carbon bonds that constitute alkanes exhibit a high degree of rotational mobility, allowing for substantial bending of the alkane chain. Conversely, the extended alkenes, comprising exclusively cis double bonds, are subject to rotationally restricted structures, giving rise to a rigid fiber configuration that differs from that of alkane chains. The binding energy between pairs of long chains has been demonstrated to be a reliable indicator of the formation of long tubular structures. These structures, with an average length of 50 Å, are formed by the lateral aggregation of dimers. The subsequent connection of these dimers by hydrophobic tails results in the formation of nanofibers.

**Figure 4:**
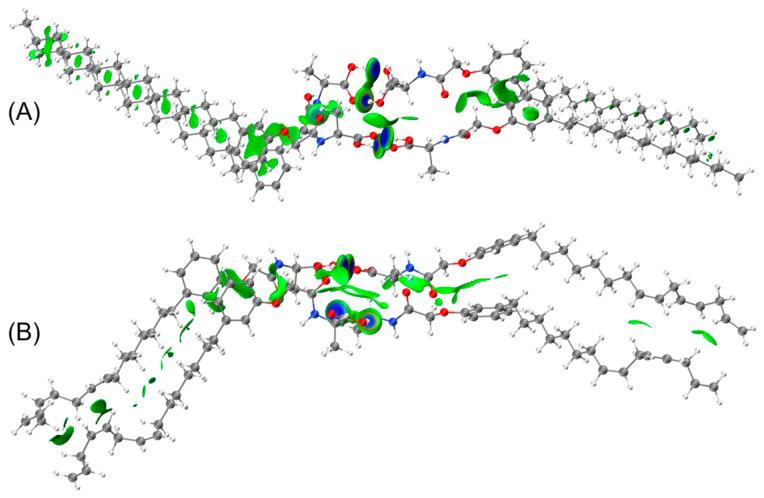
The DFT interaction model of tetramers for **PALAH** (**A**) and **CALAH** (**B**). Gray spheres correspond to carbon atoms, white spheres to hydrogen atoms, red spheres to nitrogen atoms, and blue spheres to oxygen atoms. The green cloud map represents van der Waals forces, and the blue cloud map represents hydrogen bonds.

The IGMH diagram (see Figure 4) offers a more vivid illustration of the weak interactions, i.e., van der Waals forces, between the benzene rings and alkyl chains [34,35]. The green area between the two molecules is obvious, while the blue hydrogen bond electron cloud is concentrated at the amide bonds and carboxyl groups.

### 2.5. Application of Characteristics as a Surfactant

**Figure 5:**
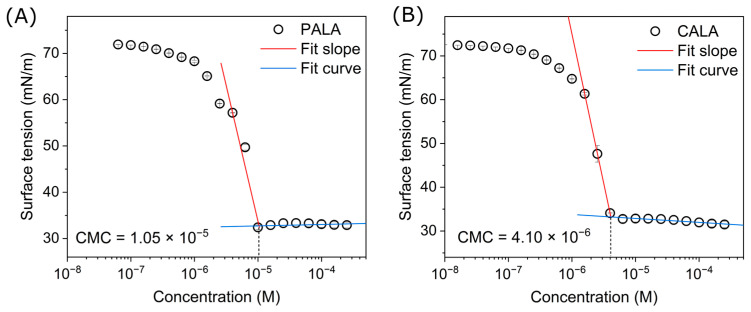
The plots of surface tension vs. concentration for **PALA** (**A**) and **CALA** (**B**).

These two amphiphilic compounds exhibit surprising characteristics; therefore, their performance as surfactants was studied using their carboxylate salts [36,37]. Initially, the subjects’ capacity to diminish surface activity was assessed. Figure 5 presents the concentration-dependent surface tension curves of the **PALA** and **CALA** solutions, as measured utilizing the ring method [38,39]. For **PALA**, when the concentration is high, the surface tension reaches a minimum value of 32.4 mN/m and remains essentially constant. As the concentration gradually decreases from high levels, the surface tension increases sharply when the concentration drops to 1.05 × 10^−5^ mol/L. Consequently, the CMC of **PALA** was determined to be 1.05 × 10^−5^ mol/L. In a similar vein, the CMC of **CALA** was determined to be 4.10 × 10^−6^ mol/L.

The CMC data for **PALA** and **CALA** surfactants, corresponding minimum surface tension (γ_CMC_) and the effectiveness of surface tension reduction (Π_CMC_), are listed in Table 2 [10]. The analysis of the data yielded the following conclusions: both substances demonstrate excellent surface activity, **CALA** exhibits a comparatively diminished CMC, and **PALA** possesses the capacity to reduce the surface tension of the solution. The findings indicate that **CALA** possesses a lower CMC value, suggesting that a smaller amount of the compound is necessary to attain the critical micelle concentration. It has been demonstrated that **CALA** possesses a higher γ_CMC_ value of 33.2 mN/m, while the ability of the two surfactants to reduce surface activity is indicated by the value of Π_CMC_. It is evident that **CALA** exhibits a greater ability in this regard.

According to Davies’ method, the hydrophilic–lipophilic balance (HLB) value of the two surfactants was calculated [40].

HLB = 7 + H_-COONa_ × N_-COONa_ + H_amide_ × N_amide_ + H_-O-_ × N_-O-_ + L_aromatic ring_ × N_aromatic ring_ + L_aliphatic -CH3, -CH2, -CH, = CH2, and = CH_ × N_aliphatic -CH3, -CH2, -CH, = CH2, and = CH_ = 13.7 (1)

where H, L, and N represent the contribution of each hydrophilic group, the contribution of each lipophilic group, and the number of each hydrophilic group or lipophilic group, respectively. According to Griffin’s method, it can be concluded that this surfactant is more suitable for use in detergents [41].

In addition, the foaming capacity of the samples was assessed by employing the Ross–Miles method [42]. The results indicated that the initial foam height of **PALA** was 31 mm, and after 5 min of incubation at 40 °C, the height decreased to 20 mm. At the initial stage, the foam height of **CALA** was recorded at 82 mm. After 5 min of incubation at 40 °C, a reduction in height to 59 mm was observed. It is evident that **CALA** exhibits a markedly superior propensity for foaming in comparison to **PALA**, a phenomenon that can be attributed to the presence of double bonds within the lipophilic group. The two materials exhibit comparable levels of foam stability, a property attributed to the hydrophilic groups, which contain a higher proportion of electronegative atoms, thereby increasing their propensity to form hydrogen bonds with water molecules.

### 2.6. Cell and Microbial Inhibitory Properties

**Figure 6:**
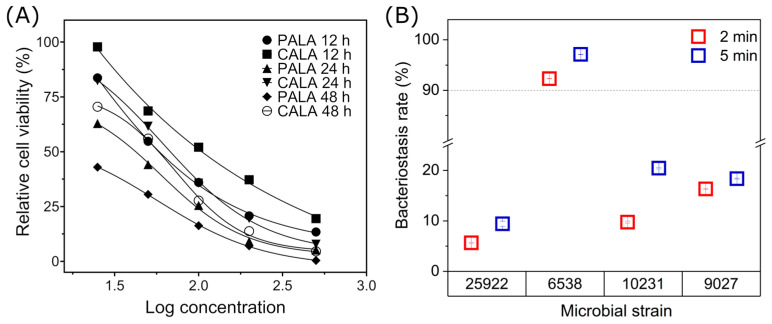
(**A**) In vitro cytotoxicity of **PALA** and **CALA** against the Hela cell line and their IC_50_ calculated by curve fitting. (**B**) Bacteriostasis rate of **PALA** and **CALA** (1 mM) against four common microorganisms. The code names definitions are as follows: ATCC 25922—*Escherichia coli*, ATCC 6538—*Staphylococcus aureus*, ATCC 10231—*Candida albicans*, ATCC 9027—*Pseudomonas aeruginosa*.

To illustrate the possible application of this surfactant as a detergent in household cleaning products, an in vitro assay was utilized to evaluate the in vitro toxicity of **PALA** and **CALA** against the HeLa cell line and L929 mouse fibroblast cells. The results of the test on L929 cells are presented in Figure S25. As illustrated in Figure 6A, the cell survival rates of the HeLa cell line were examined over time at varying concentrations (25 μg/mL, 50 μg/mL, 100 μg/mL, 200 μg/mL, and 500 μg/mL) of the two surfactants. The half maximal inhibitory concentration (IC_50_) values for **PALA** and **CALA** at 24 h were determined to be 59.9 μg/mL and 61.2 μg/mL, respectively, based on the curves in the figure. Briefly, **CALA** exhibits lower toxicity, and both **PALA** and **CALA** demonstrate toxicity levels that are significantly lower than those of the commonly used SDS (IC_50_ = 31.40 μg/mL) [36]. This result is consistent with findings for other amino acid-based surfactants [43,44].

The efficacy of bio-based surfactants and certain natural products in disrupting the established biofilms of various pathogens has been demonstrated, thereby inhibiting bacterial growth [45,46]. Therefore, an experiment was conducted to examine the effects of two surfactants against four microorganisms. As illustrated in Figure 6B, the results demonstrate the inhibitory effects of **CALA** on the four microorganisms at a concentration of 1 mM after 2 and 5 min. The inhibition rate for *Staphylococcus aureus* was over 90%, at 92.3% and 97.1%, respectively, while the inhibition rates for the other three microorganisms ranged from 5% to 21%.

### 2.7. Gas Adsorption Properties

**Figure 7:**
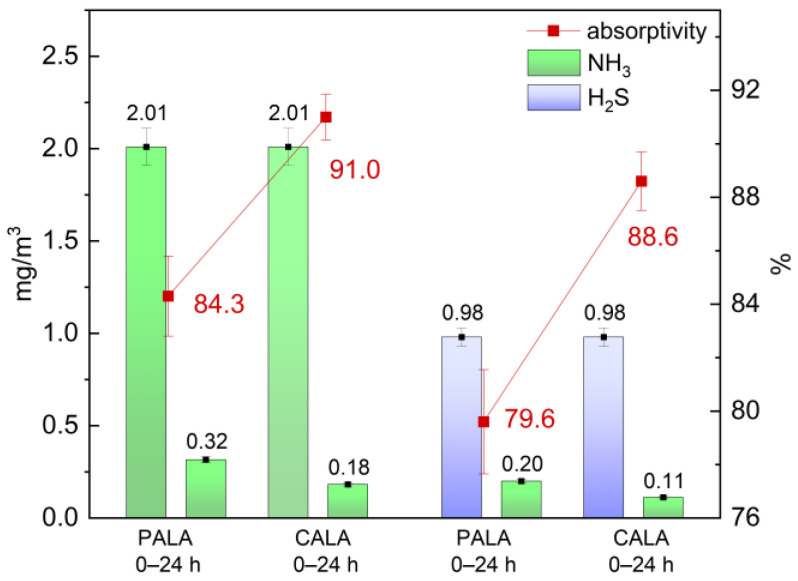
Gas adsorption for two common gas pollutants (NH_3_ and H_2_S).

The self-assembly of these molecules into nanofibers and the subsequent formation of a fibrous network structure are predicated on their unique structural characteristics. Consequently, we hypothesize that this structure contains nanoscale pores capable of adsorbing small gas molecules. In order to verify this hypothesis and explore the functionalization of the compound, experiments were conducted on the purification of gaseous pollutants. The experiments adhered to the method QB/T 2761-2024 [47]. In the experiments, the two compounds, **PALAH** and **CALAH**, were each prepared as 1 mM solutions in 100 mL of sample solution. These solutions were applied in three separate instances, with each instance involving the uniform spraying of the solution onto a separate 1 m^2^ base paper. Subsequent to a period of natural drying, the papers were placed within a 1.5 m^3^ test chamber for a duration of 24 h. Gas concentration measurements were then taken. The results of the experiments are presented in Figure 7. In comparison with the blank control group, the removal rates for NH_3_ were 84.3% and 91.0% for **PALAH** and **CALAH**, respectively, and for H_2_S, the removal rates were 79.6% and 88.6%, respectively. These results are noteworthy, as both compounds demonstrated effective removal of the two common pollutants, with better results for ammonia, and with **CALAH** exhibiting greater efficacy than **PALAH** by a margin of 6 to 9 percentage points.

## 3. Materials and Methods

### 3.1. Synthesis Materials and Characterization

Compound **1a** (Cardanol, ≥98.0%) was purchased from Guangdong Wengjiang Chemical Reagent Co., Ltd. (Shaoguan, China). **1b** (3-Pentadecylphenol (>90.0%), *L*-alanine methyl ester hydrochloride (98.0%), *N*,*N*′-dicyclohexylcarbodiimide (99.0%, DCC), 1-hydroxybenzotriazole (99.0%, HOBt), triethylamine (99.0%), potassium carbonate (99.5%), magnesium sulfate (99.0%), methanol (99.5%), acetonitrile (99.0%), and tetrahydrofuran (THF, 99.0%) were purchased from Shanghai Macklin Biochemical Co., Ltd. (Shanghai, China). Methyl chloroacetate (≥99.0%), sodium hydroxide (≥98%, pellets), were purchased from Shanghai Aladdin Biochemical Technology Co., Ltd. (Shanghai, China). Dichloromethane (DCM, ≥99.5%, Greagent), petroleum ether (60/90, Greagent), and ethyl acetate (99.9%, Adamas-beta^®^) were purchased from Shanghai Titan Scientific Co., Ltd. (Shanghai, China). All the other reagents and solvents were used as received.

^1^H and ^13^C NMR spectra were recorded on a Avance-400 spectrometer, Avance-500 spectrometer, Avance-600 spectrometer (Bruker, Billerica, MA, USA) with CDCl_3_ and DMSO-*d*_6_ as deuterated solvent.

FT-IR spectroscopy was performed using a Tensor 27 FTIR spectrometer (Bruker, Billerica, MA, USA).

High-Resolution Mass Spectrometry (HRMS) was performed using Q Exactive (Thermo Fisher, Scientific, Waltham, MA, USA).

### 3.2. Synthesis of Amphiphilic Molecules **CALAH** and **PALAH**

#### 3.2.1. Methyl Cardenolphenoxyacetate (**2a**)

In a 1000 mL flask, **1a** (cardanol (59.7 g, 200 mmol, 1.0 equiv.), potassium carbonate (55.3 g, 400 mmol, 1.1 equiv.), and methyl chloroacetate (23.9 g, 220 mmol, 2.0 equiv.) were added sequentially, followed by the addition of acetonitrile (350 mL). The mixture was heated to 82 °C and refluxed for 16 h. After the reaction was completed, the heating was stopped and the mixture was cooled to room temperature. The reaction mixture was washed with brine and extracted with dichloromethane (3 × 200 mL). The organic extract was dried over anhydrous magnesium sulfate and evaporated under reduced pressure. The crude solid was purified by flash column chromatography on silica gel using dichloromethane/petroleum ether 1:4, obtaining compound **2a** (methyl cardenolphenoxyacetate, 64.3 g. 172 mmol, 86%) as a yellow liquid.

R*_f_* = 0.55 (petroleum ether/ethyl acetate 20:1)

^1^H NMR (500 MHz, CDCl_3_, ppm) δ = 7.19 (t, *J* = 7.9 Hz, 1H, C**H**^Ph^), 6.83 (d, *J* = 7.5 Hz, 1H, C**H**^Ph^), 6.77 (t, *J* = 2.1 Hz, 1H, C**H**^Ph^), 6.74–6.65 (m, 1H, C**H**^Ph^), 5.83 (ddt, *J* = 16.5, 10.1, 6.2 Hz, 0.5H, CH_2_=C**H**), 5.49–5.31 (m, 3H, CH=C**H**), 5.14–4.94 (m, 1H, C**H**_2_=CH), 4.63 (s, 2H, O-C**H**_2_), 3.81 (s, 3H, O-C**H**_3_), 2.82 (dq, *J* = 17.6, 6.0 Hz, 2H, CH=CH-C**H**_2_-CH=CH), 2.58 (q, *J* = 10.2, 9.0 Hz, 2H, Ph-C**H**_2_), 2.09–2.00 (m, 4H, CH=CH-C**H**_2_), 1.60 (q, *J* = 7.3 Hz, 2H, C**H**_2_), 1.47–1.23 (m, 14H, C**H**_2_), 0.92 (dt, *J* = 12.5, 7.2 Hz, 2H, CH_2_-C**H**_3_).

^13^C NMR (126 MHz, CDCl_3_, ppm) δ = 169.6 (**C**=O), 157.8 (O-**C**^Ph^), 144.8 (CH_2_-**C**^Ph^), 136.8 (**C**H^C=C^), 130.4 (**C**H^C=C^), 130.1 (**C**H^C=C^), 129.9 (**C**H^C=C^), 129.8 (**C**H^C=C^), 129.3 (**C**H^C=C^, **C**H^Ph^), 128.2 (**C**H^C=C^), 128.0 (**C**H^C=C^), 127.6 (**C**H^C=C^), 126.8 (**C**H^C=C^), 122.0 (**C**H^Ph^), 115.1 (**C**H^Ph^), 114.7(**C**H^C=C^), 111.4 (**C**H^Ph^), 65.3 (O-**C**H_2_), 52.2 (O-**C**H_3_), 36.0 (Ph-**C**H_2_), 31.8 (**C**H_2_), 31.6 (**C**H_2_), 31.3 (**C**H_2_), 31.3 (**C**H_2_), 29.8 (**C**H_2_), 29.8 (**C**H_2_), 29.7 (**C**H_2_), 29.7 (**C**H_2_), 29.4 (**C**H_2_), 29.3 (**C**H_2_), 29.3 (**C**H_2_), 29.3 (**C**H_2_), 29.3 (**C**H_2_), 29.0 (**C**H_2_), 27.3 (**C**H_2_), 27.2 (**C**H_2_), 25.7 (**C**H_2_), 25.6 (**C**H_2_), 22.8 (**C**H_2_), 22.7 (**C**H_2_), 14.1 (**C**H_3_), 13.8 (**C**H_3_).

HRMS-ESI (*m*/*z*): [M_n=6_ + Na]^+^, calc. for C_24_H_34_O_3_Na, 393.2400; found: 393.2400. [M_n=4_ + Na]^+^, calc. for C_24_H_36_O_3_Na, 395.2557; found: 395.2548. [M_n=2_ + Na]^+^, calc. for C_24_H_38_O_3_Na, 397.2713; found: 397.2705.

#### 3.2.2. Cardenolphenoxyacetic Acid (**3a**)

In a 500 mL flask, **2a** (54.6 g, 147 mmol, 1.0 equiv.) and sodium hydroxide (11.8 g, 294 mmol, 2.0 equiv.) were added, followed by a mixed solvent of water and methanol (300 mL, 1:4, *v*/*v*). The reaction mixture was stirred at 25 °C for 6 h, when all solids were dissolved and stopped. The reaction was then neutralized with dilute hydrochloric acid to pH = 5. The mixture was extracted with dichloromethane (3 × 150 mL), and the organic extract was dried over anhydrous magnesium sulfate. Then the solvent was evaporated under reduced pressure to give a yellow viscous solid, which, when dried, yielded compound **3a** (cardenolphenoxyacetic acid, 49.9 g, 140 mmol, 95%).

^1^H NMR (500 MHz, CDCl_3_, ppm) δ = 6.88 (t, *J* = 7.8 Hz, 1H, C**H**^Ph^), 6.59 (d, *J* = 7.5 Hz, 1H, C**H**^Ph^), 6.51 (d, *J* = 6.9 Hz, 2H, C**H**^Ph^), 5.79 (ddt, *J* = 16.6, 10.1, 6.1 Hz, 0.5H, CH_2_=C**H**), 5.46–5.26 (m, 3H, CH_2_=C**H**), 5.09–4.91 (m, 1H, C**H**_2_=CH), 4.16 (s, 2H, O-C**H**_2_), 2.78 (dq, *J* = 17.0, 5.9 Hz, 2H, CH=CH-C**H**_2_-CH=CH), 2.31 (t, *J* = 8.1 Hz, 2H, Ph-C**H**_2_), 2.00 (tt, *J* = 14.9, 8.5 Hz, 4H, CH=CH-C**H**_2_), 1.39 (td, *J* = 14.6, 13.1, 5.8 Hz, 2H, C**H**_2_), 1.31–1.23 (m, 6H, C**H**_2_), 1.21 (m, 6H, C**H**_2_), 0.88 (dt, *J* = 11.5, 7.2 Hz, 2H, CH_2_-C**H**_3_).

^13^C NMR (126 MHz, CDCl_3_, ppm) δ = 175.0 (**C**=O), 157.5 (O-**C**^Ph^), 144.5 (CH_2_-**C**^Ph^), 136.8 (**C**H^C=C^), 130.3, 130.1 (**C**H^C=C^), 129.9 (**C**H^C=C^), 129.8 (**C**H^C=C^), 129.3 (**C**H^C=C^), 129.2 (**C**H^C=C^), 128.2 (**C**H^C=C^), 128.0(**C**H^C=C^), 127.6 (**C**H^C=C^), 126.8 (**C**H^C=C^), 121.3 (**C**H^Ph^), 115.3 (**C**H^Ph^), 114.8 (**C**H^Ph^), 114.7 (**C**H^Ph^), 111.8 (**C**H^Ph^), 66.8 (O-**C**H_2_), 35.9 (Ph-**C**H_2_), 31.8 (**C**H_2_), 31.5 (**C**H_2_), 31.4 (**C**H_2_), 29.9 (**C**H_2_), 29.8 (**C**H_2_), 29.7 (**C**H_2_), 29.5 (**C**H_2_), 29.4 (**C**H_2_), 29.3 (**C**H_2_), 29.0 (**C**H_2_), 27.3 (**C**H_2_), 27.2 (**C**H_2_), 25.7 (**C**H_2_), 25.6 (**C**H_2_), 22.8 (**C**H_2_), 22.7 (**C**H_2_), 14.1 (**C**H_3_), 13.8 (**C**H_3_).

HRMS-ESI (*m*/*z*): [M_n=6_ − H]^−^, calc. for C_23_H_31_O_3_, 355.2279; found: 355.2280. [M_n=4_ − H]^−^, calc. for C_23_H_33_O_3_, 357.2435; found: 357.2434. [M_n=2_ − H]^−^, calc. for C_23_H_35_O_3_, 359.2592; found: 359.2592.

#### 3.2.3. Methyl (Cardenolphenoxy)acetyl-*L*-alaninate (**4a**)

In a 1000 mL flask, **3a** (35.9 g, 100 mmol, 1.0 equiv.) was combined with *N*,*N*′-dicyclohexylcarbodiimide (DCC, 15.4 g, 110 mmol, 1.1 equiv.) and 1-hydroxybenzotriazole (HOBt, 16.2 g, 120 mmol, 1.2 equiv.), followed by the addition of dichloromethane (300 mL). The mixture was stirred for 0.5 h. In a separate flask, *L*-alanine methyl ester hydrochloride (15.4 g, 110 mmol, 1.1 equiv.) and triethylamine (TEA, 12.1 g, 120 mmol, 1.2 equiv.) were dissolved in tetrahydrofuran (150 mL) and reacted for 0.5 h. The two reaction mixtures were then combined and stirred at 25 °C for 24 h. The reaction mixture was washed with brine and extracted with dichloromethane (3 × 150 mL). The organic extract was dried over anhydrous magnesium sulfate and evaporated under reduced pressure. The crude solid was purified by flash column chromatography on silica gel using dichloromethane/petroleum ether/ethyl acetate 5:20:1, producing compound **4a** (methyl (cardenolphenoxy)acetyl-*L*-alaninate, 32.3 g, 72.7 mmol, 73%) as a beige, viscous liquid.

R*_f_* = 0.5 (petroleum ether/ethyl acetate 20:1)

^1^H NMR (500 MHz, CDCl_3_, ppm) δ = 7.20 (t, *J* = 7.8 Hz, 1H, C**H**^Ph^), 7.17 (d, *J* = 7.7 Hz, 1H, N**H**), 6.84 (d, *J* = 7.5 Hz, 1H, C**H**^Ph^), 6.77 (t, *J* = 2.0 Hz, 1H, C**H**^Ph^), 6.74 (dd, *J* = 8.1, 2.6 Hz, 1H, C**H**^Ph^), 5.80 (ddt, *J* = 16.5, 10.0, 6.2 Hz, 0.5H, CH_2_=C**H**), 5.47–5.28 (m, 3H, CH_2_=C**H**), 5.08–4.95 (m, 1H, C**H**_2_=CH), 4.69 (p, *J* = 7.3 Hz, 1H, NH-C**H**), 4.48 (s, 2H, O-C**H**_2_), 3.75 (s, 3H, O-C**H**_3_), 2.79 (dq, *J* = 18.2, 6.1 Hz, 2H, CH=CH-C**H**_2_-CH=CH), 2.61–2.52 (m, 2H, Ph-C**H**_2_), 2.08–1.96 (m, 3H, CH=CH-C**H**_2_), 1.60 (t, *J* = 7.6 Hz, 2H, C**H**_2_), 1.45 (d, *J* = 7.2 Hz, 3H, CH-C**H**_3_), 1.37–1.25 (m, 12H, C**H**_2_), 0.89 (dt, *J* = 12.7, 7.2 Hz, 2H, CH_2_-C**H**_3_).

^13^C NMR (126 MHz, CDCl_3_, ppm) δ = 173.0 (**C**=O), 168.0 (**C**=O), 157.3 (O-**C**^Ph^), 145.0 (CH_2_-**C**^Ph^), 136.8(**C**H^C=C^), 130.4 (**C**H^C=C^), 130.1 (**C**H^C=C^), 130.0 (**C**H^C=C^), 129.9 (**C**H^C=C^), 129.8 (**C**H^C=C^), 129.5 (**C**H^C=C^, **C**H^Ph^), 129.3 (**C**H^C=C^), 128.2 (**C**H^C=C^), 128.0 (**C**H^C=C^), 127.6 (**C**H^C=C^), 126.8 (**C**H^C=C^), 122.3 (**C**H^Ph^), 115.1 (**C**H^Ph^), 114.7 (**C**H^C=C^), 111.7 (**C**H^Ph^), 67.3 (O-**C**H_2_), 52.5 (O-**C**H_3_), 47.6 (NH-**C**H), 36.0 (Ph-**C**H_2_), 31.8 (**C**H_2_), 31.5 (**C**H_2_), 31.4 (**C**H_2_), 29.8 (**C**H_2_), 29.7 (**C**H_2_), 29.6 (**C**H_2_), 29.4 (**C**H_2_), 29.3 (**C**H_2_), 29.2 (**C**H_2_), 29.0 (**C**H_2_), 27.2 (**C**H_2_), 25.7 (**C**H_2_), 25.6 (**C**H_2_), 22.8 (**C**H_2_), 22.7 (**C**H_2_), 18.4 (**C**H_2_), 14.1 (**C**H_3_), 13.8 (**C**H_3_).

HRMS-ESI (*m*/*z*): [M_n=6_ − H]^−^, calc. for C_27_H_38_NO_4_, 440.2806; found: 440.3519. [M_n=4_ − H]^−^, calc. for C_27_H_40_NO_4_, 442.2963; found: 442.2948. [M_n=2_ − H]^−^, calc. for C_27_H_42_NO_4_, 444.3119; found: 444.3119.

#### 3.2.4. Sodium (Cardenolphenoxy)acetyl-*L*-alanine (**CALA**) and (Cardenolphenoxy)acetyl-*L*-alanine (**CALAH**)

In a 250 mL flask, **4a** (21.5 g, 48.7 mmol, 1.0 equiv.) and sodium hydroxide (2.0 g, 97.4mmol, 2.0 equiv.) were added, followed by a mixed solvent of water and methanol (200 mL, 1:4, *v*/*v*). The reaction mixture was stirred at 25 °C for 6 h, until all solids were dissolved. The solvent was evaporated under reduced pressure, methanol was added, and the solution was filtered to produce product **CALA** (sodium (cardenolphenoxy)acetyl-L-alanine, 20.5 g, 45.3 mmol, 93%). The product **CALA** was then neutralized with dilute hydrochloric acid to pH = 5 and extracted with dichloromethane (3 × 100 mL). The organic extract was dried over anhydrous magnesium sulfate. Then, the solvent was evaporated under reduced pressure to give a yellow viscous solid, which, when dried, yielded **CALAH** ((cardenolphenoxy)acetyl-*L*-alanine, 19.1 g, 44.3 mmol, 91%).

^1^H NMR (500 MHz, CDCl_3_, ppm) δ = 7.23 (d, *J* = 7.2 Hz, 1H, N**H**), 7.17 (t, *J* = 7.9 Hz, 1H, C**H**^Ph^), 6.82 (d, *J* = 7.5 Hz, 1H, C**H**^Ph^), 6.76–6.73 (m, 1H, C**H**^Ph^), 6.71 (dd, *J* = 8.2, 2.6 Hz, 1H, C**H**^Ph^), 6.50–6.42 (m, 1H, N**H**), 5.81 (ddt, *J* = 16.5, 10.0, 6.2 Hz, 0.5H, CH_2_=C**H**), 5.49–5.28 (m, 3H, CH_2_=C**H**), 5.09–4.95 (m, 1H, C**H**_2_=CH), 4.57–4.40 (m, 3H, NH-C**H**, O-C**H**_2_), 2.80 (dq, *J* = 18.3, 6.2 Hz, 2H, CH=CH-C**H**_2_-CH=CH), 2.61–2.49 (m, 2H, Ph-C**H**_2_), 2.06–1.97 (m, 3H, CH=CH-C**H**_2_), 1.57 (p, *J* = 7.6 Hz, 2H, C**H**_2_), 1.44 (d, *J* = 7.2 Hz, 3H, CH-C**H**_3_), 1.39–1.23 (m, 14H, C**H**_2_), 0.89 (dt, *J* = 13.0, 7.2 Hz, 2H, CH_2_-C**H**_3_).

^1^H NMR (600 MHz, DMSO-*d*_6_, ppm) δ = 8.21 (d, *J* = 7.3 Hz, 1H, N**H**), 7.18 (t, *J* = 7.8 Hz, 1H, C**H**^Ph^), 6.89–6.74 (m, 3H, C**H**^Ph^), 5.44–5.25 (m, 3H, CH_2_=C**H**), 5.09–4.92 (m, 1H, C**H**_2_=CH), 4.48 (d, *J* = 4.1 Hz, 2H, O-C**H**_2_), 4.25 (p, *J* = 7.2 Hz, 1H, NH-C**H**), 2.85–2.70 (m, 2H, CH=CH-C**H**_2_-CH=CH), 2.53 (t, *J* = 8.1 Hz, 2H, Ph-C**H**_2_), 2.00 (dq, *J* = 19.5, 6.7 Hz, 3H, CH=CH-C**H**_2_), 1.54 (h, *J* = 5.9, 4.5 Hz, 2H, C**H**_2_), 1.31 (d, *J* = 7.2 Hz, 3H, CH-C**H**_3_), 1.29–1.17 (m, 14H, C**H**_2_), 0.86 (q, *J* = 7.3 Hz, 2H, CH_2_-C**H**_3_).

^13^C NMR (101 MHz, CDCl_3_, ppm) δ = 176.1 (**C**=O), 169.1 (**C**=O), 157.2 (O-**C**^Ph^), 145.0 (CH_2_-**C**^Ph^), 136.8 (**C**H^C=C^), 130.4 (**C**H^C=C^), 130.1 (**C**H^C=C^), 130.0 (**C**H^C=C^), 129.9 (**C**H^C=C^), 129.8 (**C**H^C=C^), 129.5 (**C**H^C=C^), 129.3 (**C**H^C=C^), 128.2 (**C**H^C=C^), 128.1 (**C**H^C=C^), 127.6 (**C**H^C=C^), 126.9 (**C**H^C=C^), 122.3 (**C**H^Ph^), 115.1 (**C**H^Ph^), 114.7 (**C**H^C=C^), 111.8 (**C**H^Ph^), 67.1 (O-**C**H_2_), 48.6 (NH-**C**H), 36.0 (Ph-**C**H_2_), 31.8 (**C**H_2_), 31.6 (**C**H_2_), 31.4 (**C**H_2_), 29.8 (**C**H_2_), 29.7 (**C**H_2_), 29.5 (**C**H_2_), 29.4 (**C**H_2_), 29.3 (**C**H_2_), 29.0 (**C**H_2_), 27.3 (**C**H_2_), 27.2 (**C**H_2_), 25.7 (**C**H_2_), 25.6 (**C**H_2_), 25.5 (**C**H_2_), 22.8 (**C**H_2_), 22.7 (**C**H_2_), 17.8 (**C**H_2_), 14.1 (**C**H_3_), 13.8 (**C**H_3_).

HRMS-ESI (*m*/*z*): [M_n=6_ − H]^−^, calc. for C_26_H_36_NO_4_, 426.2650; found: 426.3060. [M_n=4_ − H]^−^, calc. for C_26_H_38_NO_4_, 428.2806; found: 442.2948. [M_n=2_ − H]^−^, calc. for C_26_H_40_NO_4_, 430.2963; found: 430.3374.

IR (ATR, ṽ) = 3390, 3321, 2924, 2848, 2653, 2609, 1724, 1627, 1544, 1454, 1421, 1298, 1244, 1204, 1110, 995, 908, 764, 691, 616, 590, 558 cm^−1^.

#### 3.2.5. Methyl 3-Pentadecylphenoxyacetate (**2b**)

In a 500 mL flask, **1b** (3-pentadecylphenol, 30.5 g, 100 mmol, 1.0 equiv.), potassium carbonate (27.6 g, 200 mmol, 1.1 equiv.), and methyl chloroacetate (11.9 g, 110 mmol, 2.0 equiv.) were added sequentially, followed by the addition of acetonitrile (250 mL). The mixture was heated to 82 °C and refluxed for 16 h. After the reaction was completed, the heating was stopped and the mixture was cooled to room temperature. The reaction mixture was washed with brine and extracted with dichloromethane (3 × 100 mL). The organic extract was dried over anhydrous magnesium sulfate and evaporated under reduced pressure. The crude solid was purified by flash column chromatography on silica gel using dichloromethane/petroleum ether 1:4, providing compound **2b** (methyl 3-pentadecylphenoxyacetate, 29.8 g. 79.2 mmol, 79%) as a white solid.

R*_f_* = 0.55 (dichloromethane/petroleum ether 1:1).

^1^H NMR (400 MHz, CDCl_3_, ppm) δ = 7.19 (t, *J* = 7.9 Hz, 1H, C**H**^Ph^), 6.82 (d, *J* = 7.6 Hz, 1H, C**H**^Ph^), 6.77 (d, *J* = 2.5 Hz, 1H, C**H**^Ph^), 6.70 (dd, *J* = 8.2, 2.6 Hz, 1H, C**H**^Ph^), 4.62 (s, 2H, O-C**H**_2_), 3.80 (s, 3H, O-C**H**_3_), 2.58 (t, *J* = 7.8 Hz, 2H, Ph-C**H**_2_), 1.61 (p, *J* = 7.1 Hz, 2H, C**H**_2_), 1.36–1.22 (m, 24H, C**H**_2_), 0.90 (t, *J* = 6.6 Hz, 3H, CH2-C**H**_3_).

^13^C NMR (101 MHz, CDCl_3_, ppm) δ = 169.5 (**C**=O), 157.9 (O-**C**^Ph^), 144.8 (CH2-**C**^Ph^), 129.3 (**C**H^Ph^), 122.0 (**C**H^Ph^), 115.1 (**C**H^Ph^), 111.4 (**C**H^Ph^), 65.3 (O-**C**H_2_), 52.2 (O-**C**H_3_), 36.0 (Ph-**C**H_2_), 32.0 (**C**H_2_), 31.4 (**C**H_2_), 29.8 (3C, **C**H_2_), 29.8 (3C, **C**H_2_), 29.7 (**C**H_2_), 29.6 (**C**H_2_), 29.4 (**C**H_2_), 29.4 (**C**H_2_), 22.8 (**C**H_2_), 14.2 (**C**H_3_).

HRMS-ESI (*m*/*z*): [M − H]^−^, calc. for C_23_H_37_O_3_, 361.2748; found: 361.2785.

#### 3.2.6. 3-Pentadecylphenoxyacetic Acid (**3b**)

In a 500 mL flask, **2b** (26.4 g, 70.0 mmol, 1.0 equiv.) and sodium hydroxide (5.6 g, 140 mmol, 2.0 equiv.) were added, followed by a mixed solvent of water and methanol (250 mL, 1:4, *v*/*v*). The reaction mixture was stirred at 25 °C for 6 h, when all solids were dissolved and stopped. The reaction was then neutralized with dilute hydrochloric acid to pH = 5. The mixture was extracted with dichloromethane (3 × 100 mL), and the organic extract was dried over anhydrous magnesium sulfate. Then, the solvent was evaporated under reduced pressure to give a white solid, which dried to yield compound **3b** (3-pentadecylphenoxyacetic acid, 24.1 g, 66.5 mmol, 95%).

^1^H NMR (500 MHz, CDCl_3_, ppm) δ = 7.18 (t, *J* = 7.9 Hz, 1H, C**H**^Ph^), 6.83 (d, *J* = 7.6 Hz, 1H, C**H**^Ph^), 6.75 (s, 1H, C**H**^Ph^), 6.71 (dd, *J* = 8.2, 2.6 Hz, 1H, C**H**^Ph^), 4.61 (s, 2H, O-C**H**_2_), 2.56 (t, *J* = 7.8 Hz, 2H, Ph-C**H**_2_), 1.58 (p, *J* = 7.3 Hz, 2H, C**H**_2_), 1.33–1.20 (m, 24H, C**H**_2_), 0.88 (t, *J* = 6.8 Hz, 3H, CH_2_-C**H**_3_).

^13^C NMR (126 MHz, CDCl_3_, ppm) δ = 173.9 (**C**=O), 157.4 (O-**C**^Ph^), 145.0 (CH_2_-**C**^Ph^), 129.3 (**C**H^Ph^), 122.3 (**C**H^Ph^), 115.2 (**C**H^Ph^), 111.6 (**C**H^Ph^), 65.3 (O-**C**H_2_), 36.0 (Ph-**C**H_2_), 32.0 (**C**H_2_), 31.3 (**C**H_2_), 29.7 (4C, **C**H_2_), 29.7 (2C, **C**H_2_), 29.6 (**C**H_2_), 29.6 (**C**H_2_), 29.4 (2C, **C**H_2_), 22.7 (**C**H_2_), 14.1 (**C**H_3_).

HRMS-ESI (*m*/*z*): [M − H]^−^, calc. for C_23_H_37_O_3_, 361.2748; found: 361.2785.

#### 3.2.7. Methyl (3-Pentadecylphenoxy)acetyl-*L*-alaninate (**4b**)

In a 500 mL flask, **3b** (18.1 g, 50.0 mmol, 1.0 equiv.) was combined with *N*,*N*′-dicyclohexylcarbodiimide (DCC, 11.3 g, 55.0 mmol, 1.1 equiv.) and 1-hydroxybenzotriazole (HOBt, 8.11 g, 60.0 mmol, 1.2 equiv.), followed by the addition of dichloromethane (200 mL). The mixture was stirred for 0.5 h. In a separate flask, *L*-alanine methyl ester hydrochloride (7.68 g, 55.0 mmol, 1.1 equiv.) and triethylamine (TEA, 6.07 g, 60.0 mmol, 1.2 equiv.) were dissolved in tetrahydrofuran (100 mL) and reacted for 0.5 h. The two reaction mixtures were then combined and stirred at 25 °C for 18 h. The reaction mixture was washed with brine and extracted with dichloromethane (3 × 100 mL). The organic extract was dried over anhydrous magnesium sulfate and evaporated under reduced pressure. The crude solid was purified by flash column chromatography on silica gel using dichloromethane/petroleum ether/ethyl acetate 5:20:1, providing compound **4b** (methyl (3-pentadecylphenoxy)acetyl-*L*-alaninate, 14.5 g, 32.5 mmol, 65%) as a pale-yellow solid.

R*_f_* = 0.5 (petroleum ether/ethyl acetate 20:1).

^1^H NMR (400 MHz, CDCl_3_, ppm) δ = 7.22–7.14 (m, 2H, C**H**^Ph^, N**H**), 6.83 (d, *J* = 7.6 Hz, 1H, C**H**^Ph^), 6.79–6.70 (m, 2H, C**H**^Ph^), 4.69 (p, *J* = 7.3 Hz, 1H, NH-C**H**), 4.48 (s, 2H, O-C**H**_2_), 3.74 (s, 3H, O-C**H**_3_), 2.57 (t, *J* = 7.8 Hz, 2H, Ph-C**H**_2_), 1.59 (p, *J* = 7.2 Hz, 2H, C**H**_2_), 1.45 (d, *J* = 7.2 Hz, 3H, CH-C**H**_3_), 1.35–1.18 (m, 24H, C**H**_2_), 0.87 (t, *J* = 6.7 Hz, 3H, CH_2_-C**H**_3_).

^13^C NMR (101 MHz, CDCl_3_, ppm) δ = 173.0 (**C**=O), 168.0 (**C**=O), 157.3 (O-**C**^Ph^), 145.1 (CH_2_-**C**^Ph^), 129.5 (**C**H^Ph^), 122.4 (**C**H^Ph^), 115.1 (**C**H^Ph^), 111.7 (**C**H^Ph^), 67.3 (O-**C**H_2_), 52.5 (O-**C**H_3_), 47.6 (NH-**C**H), 36.0 (Ph-**C**H_2_), 32.0 (**C**H_2_), 31.4 (**C**H_2_), 29.7 (2C, **C**H_2_), 29.7 (2C, **C**H_2_), 29.7 (C, **C**H_2_), 29.6 (**C**H_2_), 29.6 (**C**H_2_), 29.4 (**C**H_2_), 29.4 (**C**H_2_), 22.7 (**C**H_2_), 18.4 (**C**H_2_), 14.1 (**C**H_3_).

HRMS-ESI (*m*/*z*): [M + Na]^+^, calc. for C_27_H_45_NO_4_Na, 470.3241; found: 470.3275.

#### 3.2.8. Sodium (3-Pentadecylphenoxy)acetyl-*L*-alanine (**PALA**) and (3-Pentadecylphenoxy)acetyl-*L*-alanine (**PALAH**)

In a 250 mL flask, **4b** (11.2 g, 25.0 mmol, 1.0 equiv.) and sodium hydroxide (2.0 g, 50 mmol, 2.0 equiv.) were added, followed by a mixed solvent of water and methanol (125 mL, 1:4, *v*/*v*). The reaction mixture was stirred at 25 °C for 6 h, when all solids were dissolved and stopped. The solvent was evaporated under reduced pressure, methanol was added, and the solution was filtered to obtain product **PALA** (sodium (3-pentadecylphenoxy)acetyl-*L*-alanine, 10.8 g, 23.8 mmol, 95%). **PALA** was then neutralized with dilute hydrochloric acid to pH = 5. The mixture was extracted with dichloromethane (3 × 100 mL), and the organic extract was dried over anhydrous magnesium sulfate. Then, the solvent was evaporated under reduced pressure to give a white solid, which, when dried, yielded **PALAH** ((3-pentadecylphenoxy)acetyl-*L*-alanine, 10.1 g, 23.3 mmol, 93%).

^1^H NMR (400 MHz, CDCl_3_, ppm) δ = 7.22–7.13 (m, 2H, C**H**^Ph^, N**H**), 6.85 (d, *J* = 7.5 Hz, 1H, C**H**^Ph^), 6.80–6.72 (m, 2H, C**H**^Ph^), 4.71 (t, *J* = 7.5 Hz, 1H, NH-C**H**), 4.53 (s, 2H, O-C**H**_2_), 2.58 (t, *J* = 7.9 Hz, 2H, Ph-C**H**_2_), 1.63–1.56 (m, 3H, CH-C**H**_3_), 1.52 (d, *J* = 7.3 Hz, 2H, C**H**_2_), 1.38–1.10 (m, 24H, C**H**_2_), 0.88 (t, *J* = 6.7 Hz, 3H, CH_2_-C**H**_3_).

^1^H NMR (600 MHz, DMSO-*d*_6_, ppm) δ = 12.67 (s, 1H, COO**H**), 8.30 (d, *J* = 7.5 Hz, 1H, N**H**), 7.18 (t, *J* = 7.8 Hz, 1H, C**H**^Ph^), 6.84–6.73 (m, 3H, C**H**^Ph^), 4.54–4.44 (m, 2H, O-C**H**_2_), 4.31 (p, *J* = 7.3 Hz, 1H, NH-C**H**), 2.53 (t, *J* = 7.6 Hz, 2H, Ph-C**H**_2_), 1.55 (p, *J* = 7.1 Hz, 2H, C**H**_2_), 1.33 (d, *J* = 7.3 Hz, 3H, CH-C**H**_3_), 1.30–1.19 (m, 24H, C**H**_2_), 0.86 (t, *J* = 6.9 Hz, 3H, CH_2_-C**H**_3_).

^13^C NMR (101 MHz, CDCl_3_, ppm) δ = 176.1 (**C**=O), 168.8 (**C**=O), 157.2 (O-**C**^Ph^), 145.2 (CH_2_-**C**^Ph^), 129.5 (**C**H^Ph^), 122.5 (**C**H^Ph^), 115.1 (**C**H^Ph^), 111.8 (**C**H^Ph^), 67.2 (O-**C**H_2_), 47.8 (NH-**C**H), 36.0 (Ph-**C**H_2_), 32.0 (**C**H_2_), 31.4 (**C**H_2_), 29.7 (6C, **C**H_2_), 29.6 (**C**H_2_), 29.4 (2C, **C**H_2_), 22.7 (**C**H_2_), 18.1 (**C**H_2_), 14.2 (**C**H_3_).

HRMS-ESI (*m*/*z*): [M − H]^−^, calc. for C_26_H_42_NO_4_, 432.3119; found: 432.3119.

IR (ATR, ṽ) = 3394, 2917, 2851, 2608, 1727, 1627, 1547, 1452, 1422, 1250, 1203, 1145, 1160, 1067, 999, 904, 868, 765, 692, 629, 588, 556 cm^−1^.

### 3.3. Morphological Structures

In order to discern the disparities in morphology between the two structures, a scanning electron microscope (SEM) was utilized, Tescan Mira Lms (Libušín, Czech Republic). The samples were dispersed in a mixture of water and ethanol (V_water_: V_ethanol_ = 4:1) to prepare the samples, and the sample surfaces were treated with gold spraying before testing with the instrument.

The polarimeter used was a Krüss P8000 (A. Krüss Optronic GmbH, Hamburg, Germany), at 20 °C, the sodium D line was 589.0 nm.

### 3.4. ^1^H NMR Self-Assembled Structures

The variable temperature ^1^H NMR spectra were recorded on an Avance-600 spectrometer (Bruker, Billerica, MA, USA) with DMSO-*d*_6_ as deuterated solvent.

### 3.5. DFT Calculations

All DFT calculations have been carried out by the latest version of ORCA quantum chemistry software (Version 6.1.0) [34].

For geometry optimization calculations, the corrected version of the r2SCAN exchange-correlation functional proposed by Grimme (so-called r2SCAN-3c) [48] was adopted.

The SMD implicit solvation model [33] was used to account for the solvation effect. Then, the interaction energy between two molecules of **CALAH**/**PALAH** was calculated by the following formula:

E_bind_ = E_complex_ − (E_partA_ + E_partB_ +…) (2)

The IGMH (independent gradient model based on Hirshfeld partition) analysis [49] was performed by the Multiwfn software (Version 3.8) [32,50]. The visualization of IGMH was rendered by VMD [35].

### 3.6. Application Characteristics as Surfactant

The surface tension meter used was the Attension Sigma 700 (Biolin Scientific AB, Västra Frölunda, Sweden).

According to Davies’ method, the HLB value of the two surfactants was calculated [40].

HLB = 7 + Σ H + Σ L(3)

### 3.7. Cell and Microbial Inhibitory Properties

Fetal bovine serum (FBS) and trypsin-EDTA were purchased from Gibco (Thermo Fisher Scientific Inc., Waltham, MA, USA). PBS was purchased from Boster Biological Technology Co., Ltd. (Wuhan, China). CCK8 was purchased from MEC Medical Ltd. (Hertford, UK). CO_2_ Incubator was SCO6WE (Shel Lab, Cornelius, OR, USA). The laminar flow cabinet was SW-CJ-1F (Suzhou Hjclean Tech, Suzhou, China). The MicroplateReader was EPOCH2 (Bio Tek, Winooski, VT, USA). L929 cell and HeLa cell were purchased from iCell bioscience Inc. (Shanghai, China), Culture medium was iCell-m026-001b and MEM (iCell-0012).

Experimental procedures: cell thawing.

Turn on the water bath and adjust the temperature to 37 °C. Remove the cells stored at −80 °C, quickly place them in the water bath, and shake vigorously until the cells are completely thawed. Remove the cryopreservation fiber from the water bath. Take a 15 mL sterile centrifuge tube, add 7 mL of culture medium, then add the cells from the cryopreservation fiber. Centrifuge at 1000 rpm at room temperature for 5 min. Take a new 25 cm^2^ cell culture flask, add 5 mL of culture medium containing 10% FBS. Discard the supernatant from the centrifuge fiber, retain the pellet, and add it entirely to the culture flask. Incubate under 5% CO_2_ and at 37 °C for 8 h, then remove all the culture medium and add 5 mL of fresh medium containing 10% FBS. Continue culturing until the cells cover the entire culture flask, then proceed with the experiment.

Control group: cells cultured in complete medium.

Treatment group: Cells cultured in samples of different concentrations (HeLa cells 25 μg/mL, 50 μg/mL, 100 μg/mL, 200 μg/mL, and 500 μg/mL). The stock solution: 0.5 mM/L; L929 cell 10 μg/mL, and 20 μg/mL.

Blank group: only medium and CCK8.

Specific steps: Before plating, remove the medium from the culture flasks, wash once with PBS, and add 0.25% trypsin to digest the cells. After removing the trypsin, add medium containing 0.25% trypsin to digest the cells. Count the L929 cells and inoculate them into a 96-well plate at a density of 1 × 10^4^ cells per well. Incubate at 37 °C in a 5% CO_2_ atmosphere for 12 h. Remove the original medium and replace it with the same concentration at 200 μL per well, and continue culturing at 37 °C in a constant temperature incubator for 12 h, 24 h, and 72 h. After removing the medium, wash each well three times with PBS solution, and add 100 μL per well of medium containing 10% CCK8 reagent. Incubate at 37 °C in a 5% CO_2_ incubator for 2 h. Measure the absorbance at 450 nm using a microplate reader.

Set up a Blank group as a background control (containing only CCK8 and complete medium). The formula for calculating the cell survival rate is as follows:

Relative cell viability (%) = (A sample − A blank)/(A control − A blank) × 100%(4)

The microbiology analysis reports were provided by Gmicro testing (Guangdong detection center of microbiology, Guangzhou, China). **PALA** Report Number 2025FM10864R01, Verification Code BM5R74CK. **CALA** Report Number 2025FM10863R01, Verification Code XL5PUQS3.

### 3.8. Gas Adsorption Properties

The test was conducted in accordance with QB/T 2761-2024 standard. Testing was conducted using a test chamber with a volume of 1.5 m^3^ and dimensions of 0.9 × 0.9 × 1.85 m.

The gas concentration was detected by Ultraviolet-visible spectrophotometer TU-1810, Beijing Purkinje GENERAL Instrument Co., Ltd. (Beijing, China). The removal rate of gaseous pollutants by passive purification products was calculated using the slow-release method according to the following formula:

(5) $$y\;=\;\frac{C_{A}-C_{B}}{C_{A}}$$

where y is the removal rate, C_A_ is the pollutant concentration value in the blank test chamber (mg/m^3^), and C_B_ is the pollutant concentration value in the sample test chamber (mg/m^3^).

Sample preparation: The passive purification product is sprayed three times on three appropriately sized base papers (requiring inert materials) using a small spray pump to form a fine mist. After the first spray dries, apply the second coat, and similarly for the third coat.

Procedure: Hang the untreated base paper in blank test chamber A, and hang the base paper coated with the sprayed material in test chamber B. Place containers holding the prepared pollutant release sources (glass containers) in test chambers A_1_ and B_2_. Immediately close the chamber doors. Activate the fans in both chambers and stir for 1 min to ensure uniform mixing of the chamber air with the pollutants released from the sources, then simultaneously shut off the fans. Air sampling is conducted in the blank chamber to determine the initial concentration C_0_ of air pollutants in the blank chamber. After 24 h, air pollutant concentrations in both chambers are sampled and analyzed, yielding the concentration values in chambers A and B, denoted as C_A_ and C_B_.

## 4. Conclusions

In summary, this study successfully synthesized two amphiphilic compounds derived from cashew nut shell liquid, along with their corresponding sodium salts, and demonstrated their significant potential as surfactants. The morphological characteristics of the amphiphilic compounds, which self-assemble into nanofibers and network structures, were investigated using SEM. The self-assembly processes and the morphological differences between the two molecules were further studied through temperature-dependent NMR and DFT calculations, which made it possible to explain the reasons for their morphological formation and structural differences.

Subsequently, the basic properties of the sodium salts as surfactants were investigated, including their CMC values, surface tension, HLB value, and foam formation properties, demonstrating that both compounds are suitable for use as surfactants in subsequent applications. In vitro cytotoxicity tests were against HeLa and L929 cells conducted on the two surfactants, and their IC_50_ values were obtained by fitting the experimental data of the HeLa cells with varying concentrations, demonstrating that the two bio-based surfactants have low toxicity. Additionally, they exhibit antibacterial effects against *Staphylococcus aureus* and have potential applications in personal care and cosmetics. Finally, gas adsorption experiments were conducted for ammonia and sulfur dioxide to take advantage of the nano structural characteristics of the self-assembled systems, revealing efficient removal capabilities for both gaseous pollutants.

The findings of this study highlight the promising potential of renewable resource-derived, supramolecular self-assembling compounds regarding their application in areas such as daily cleaning, disinfection, and environmental gas adsorption.

## Figures and Tables

**Table 1:** The interaction energy between dimer/tetramer of **PALAH/CALAH**.

Molecular	kJ/mol	eV
**PALAH** dimer	−37.30	−0.39
**PALAH** tetramer	−206.91	−2.14
**CALAH** dimer	−31.88	−0.33
**CALAH** tetramer	−216.99	−2.25

**Table 2:** Surface properties of benzoxazine surfactants **PALA** and **CALA** at 25 °C and pH = 9.0.

Surfactants	CMC (mol/L)	γ_CMC_ (mN/m)	Π_CMC_ ^1^ (mN/m)
**PALA**	1.05 × 10^−5^	32.5	39.5
**CALA**	4.10 × 10^−6^	33.2	38.8

^1^ Π_CMC_ = γ_0_ − γ_CMC_, where γ_0_ was the surface tension of pure water and γ_CMC_ was the surface tension at CMC.

## Data Availability

Data is contained within the article and Supplementary Materials.

## Supplementary Materials

The following supporting information can be downloaded at: https://www.mdpi.com/article/10.3390/molecules30204119/s1, Figures S1–S18: ^1^H NMR or ^13^C NMR spectra recorded for molecules; Figures S19 and S20: IR spectra recorded for **PALAH**, **CALAH**; Figures S21–S23: 2D NMR spectra recorded for **CALAH**; Figure S24–S27: SEM images for **PALAH** and **CALAH**; Figure S27. ^1^H NMR spectra of temperature-dependent for **CALAH**; Figure S28: The DFT model of **PALAH** and **CALAH**; Figure S29: The scatter plot for **PALAH**/**CALAH**; Figure S30: In vitro cytotoxicity of **PALA** and **CALA**; Table S1: The specific rotation of **PALAH**/**CALAH**.

## Abbreviations

The following abbreviations are used in this manuscript:

CNSL	Cashew nut shell liquid
NMR	Nuclear magnetic resonance
HRMS	High-resolution mass spectrometry
FT-IR	Fourier-transform infrared spectroscopy
SEM	Scanning electron microscopy
DFT	Density functional theory
HLB	Hydrophilic lipophilic balance
CMC	Critical micelle concentration
APGs	Alkyl polyglycolides
DCC	*N*,*N*′-dicyclohexylcarbodiimide
HOBt	1-Hydroxybenzotriazole
THF	Tetrahydrofuran
Co., Ltd.	Company limited by shares
DCM	Dichloromethane
R*_f_*	Retention factor value
r.t.	Room temperature
mol	Mole
M	Mole per liter
CDCl_3_	Chloroform
Ph	Phenyl group
*J*	Coupling constant
t	Triplet
s	Singlet
q	Quartet
m	Multiplet
dd	Double doublet
dq	Double quartet
ddt	Double double triplet
pH	Potential of hydrogen
h	Hour
g	Gram
mL	Milliliter
mM	Millimole per liter
mg	Milligram
mmol	Millimole
equiv.	Equivalents
DMSO	Dimethyl sulfoxide
MHz	Mega Hertz
cm^−1^	Reciprocal centimeter
°C	Degree Celsius
ppm	Parts per million
μm	Micrometer
calc.	Calculated
Å	Angstrom
kJ	Kilojoule
TEA	Triethylamine
ESI	Electrospray ionization
*v*/*v*	Volume to Volume Ratio
δ	Chemical shift
ATR	Attenuated Total Reflectance
[α]	Specific optical rotation
EtOH	Ethanol
c	Concentration
SMD	Solvation model density
IGMH	Independent gradient model based on Hirshfeld partition
E	Energy
RDG	Reduced density gradient
CO_2_	Carbon dioxide
CCK8	Cell Counting Kit-8
PBS	Fetal bovine serum
EDTA	Ethylenediaminetetraacetic Acid
L929	NCTC clone 929
MEM	Minimum essential medium
rpm	Revolutions per minute
PBS	Phosphate-buffered saline
eV	Electron volt
SDS	Sodium dodecyl sulfate
IC_50_	Half maximal inhibitory concentration
NH_3_	Ammonia
H_2_S	Hydrogen sulfide
