# Supplementary material for: Nanofiber Networks from Self-Assembling Cardanol Amphiphiles: Toward Renewable Multifunctional Surfactants
Source: Molecules. 2025 Oct 17;30(20):4119. doi: 10.3390/molecules30204119 (PMC12565755; doi:10.3390/molecules30204119)
Supplement: Supplementary file 1 [file molecules-30-04119-s001.zip › molecules-3877861-supplementary.pdf]

molecules

MDPI

Supplementary Materials

Synthesis of amphiphilic molecules

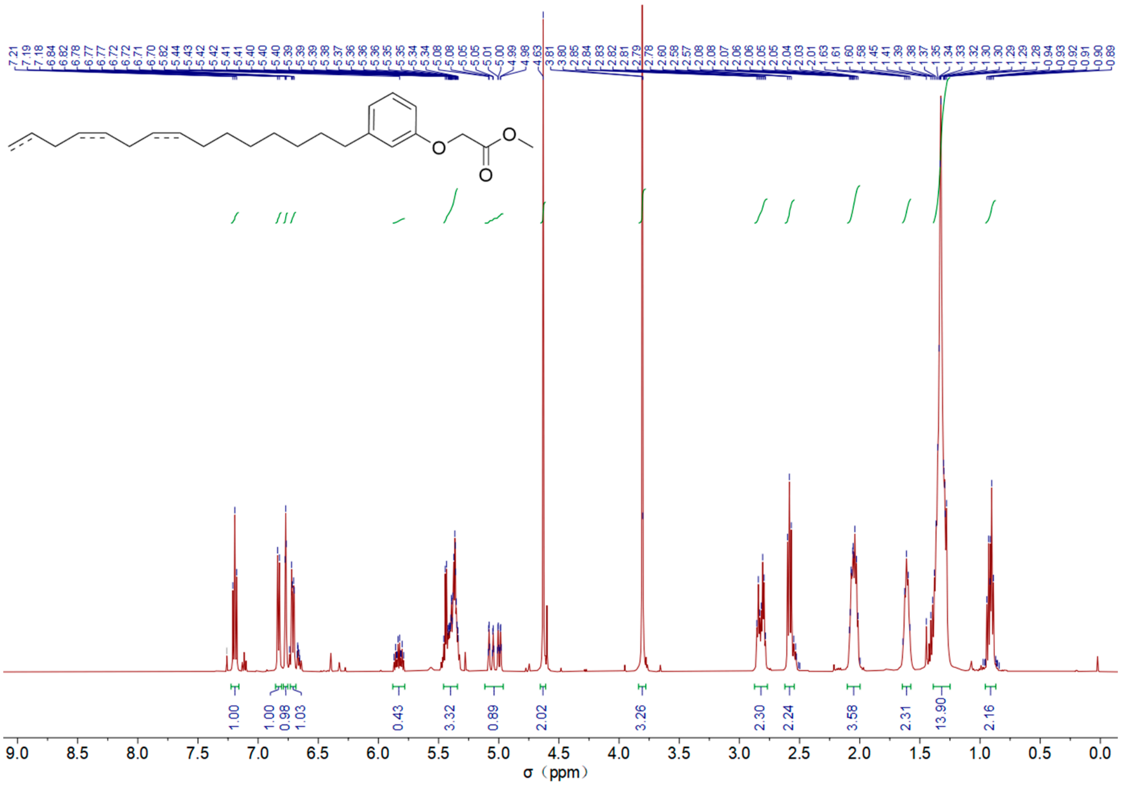

Figure S1 ^{1}H NMR spectra recorded (500 MHz, $CDCl_3$, r.t.) for **2a**

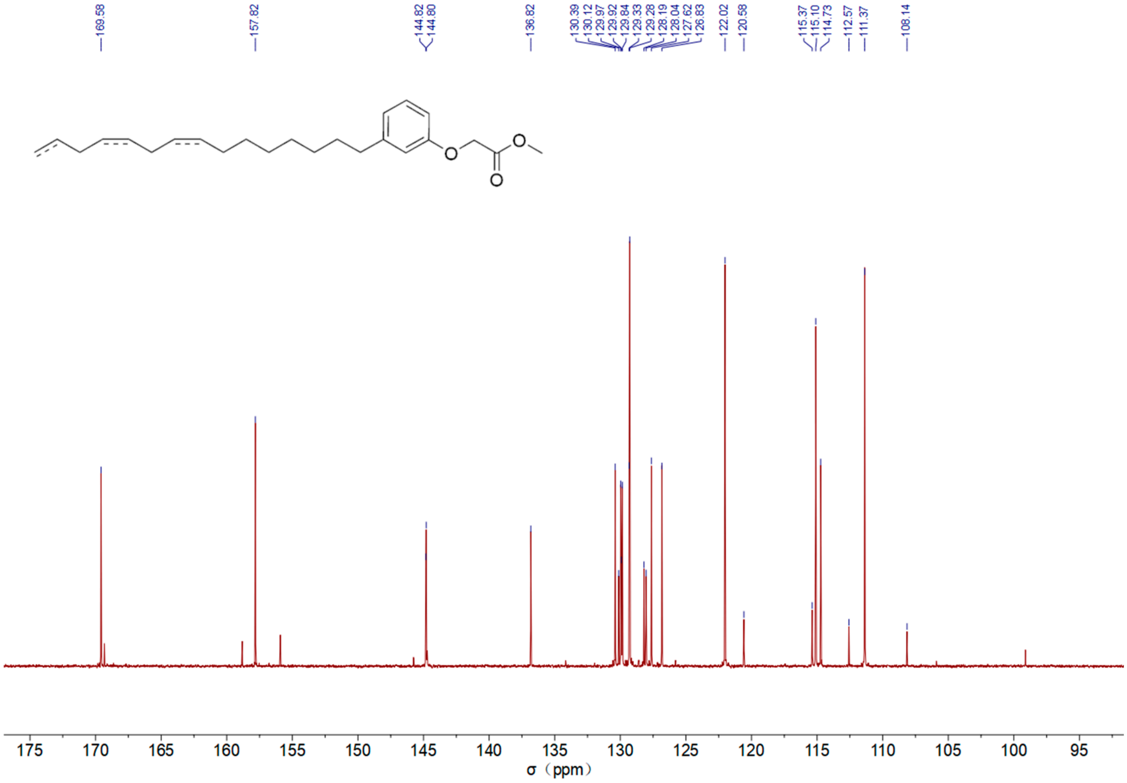

Figure S2 ^{1}H NMR spectra recorded (126 MHz, $CDCl_3$, r.t.) for **2a**

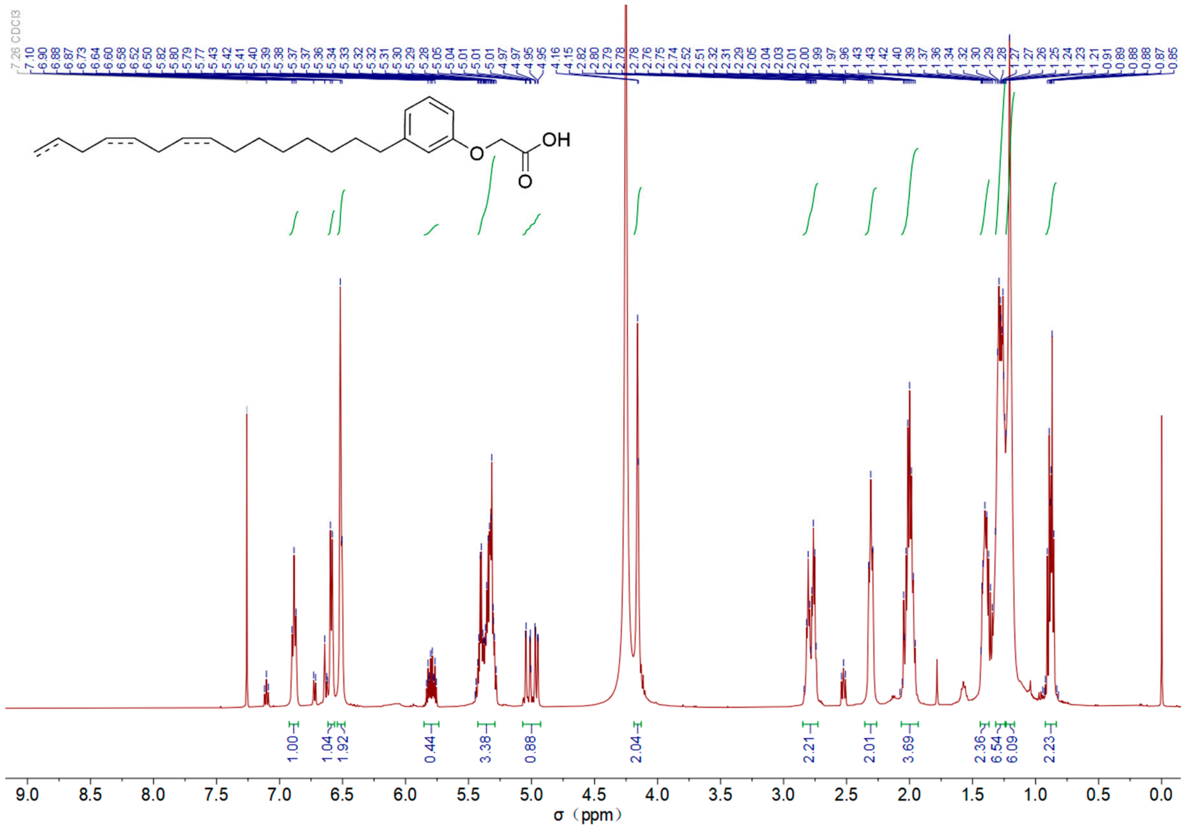

Figure S3 ^{1}H NMR spectra recorded (500 MHz, $CDCl_3$, r.t.) for **3a**

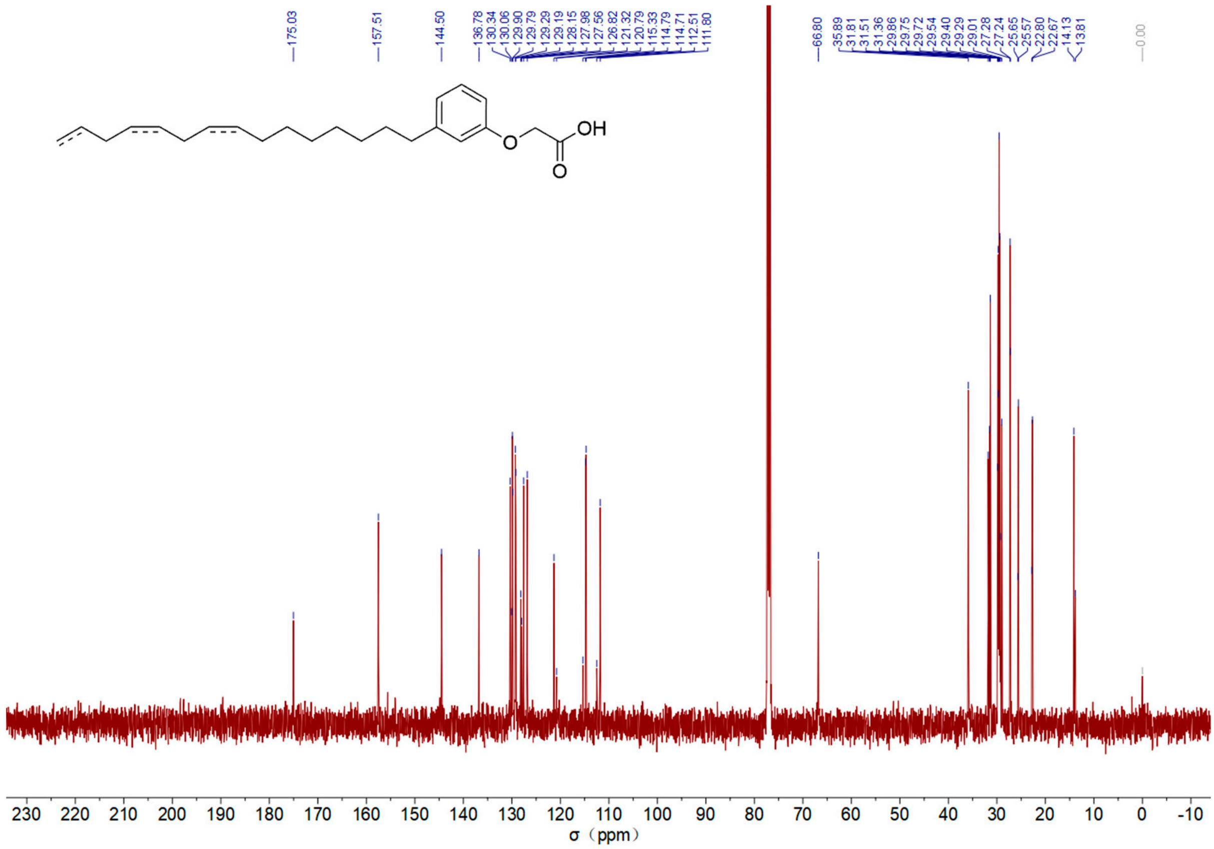

Figure S4 ^{13}C NMR spectra recorded (126 MHz, $CDCl_3$, r.t.) for **3a**

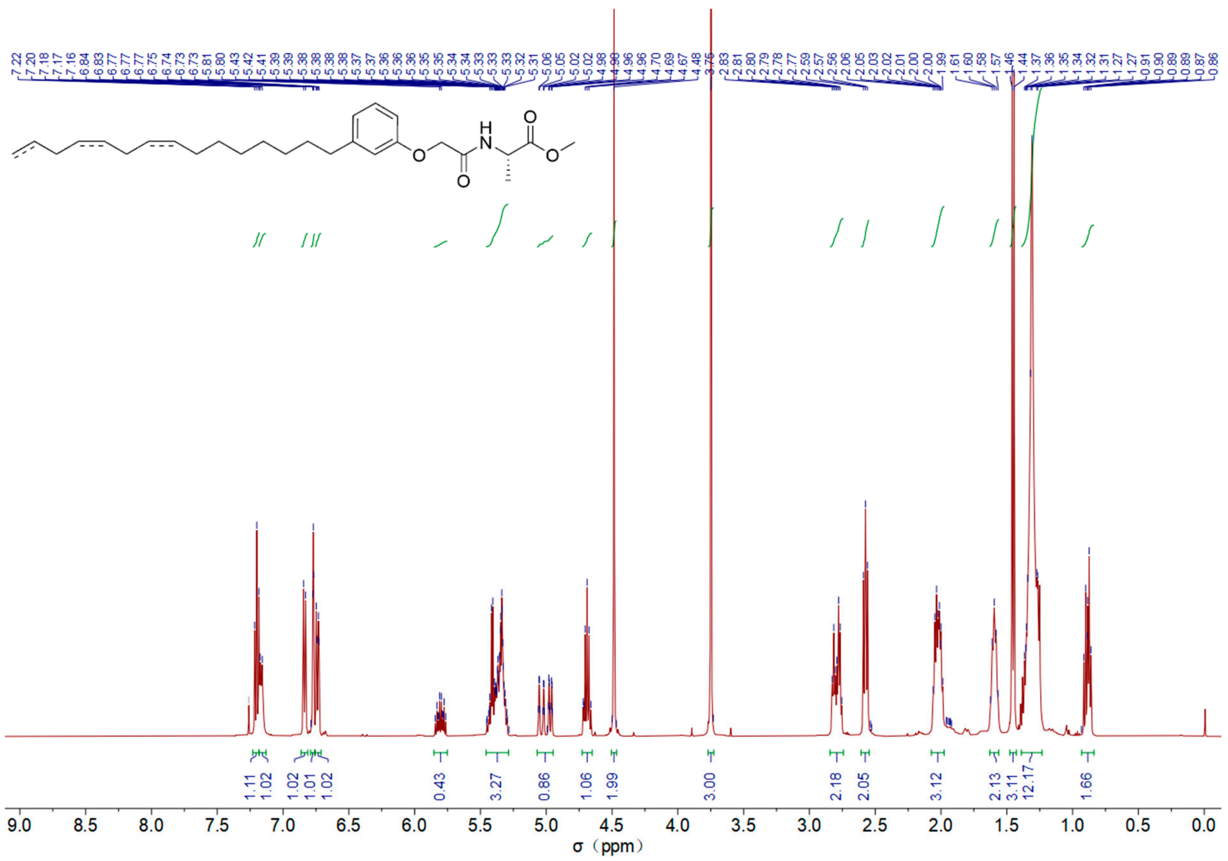

Figure S5 ^{1}H NMR spectra recorded (500 MHz, $CDCl_3$, r.t.) for **4a**

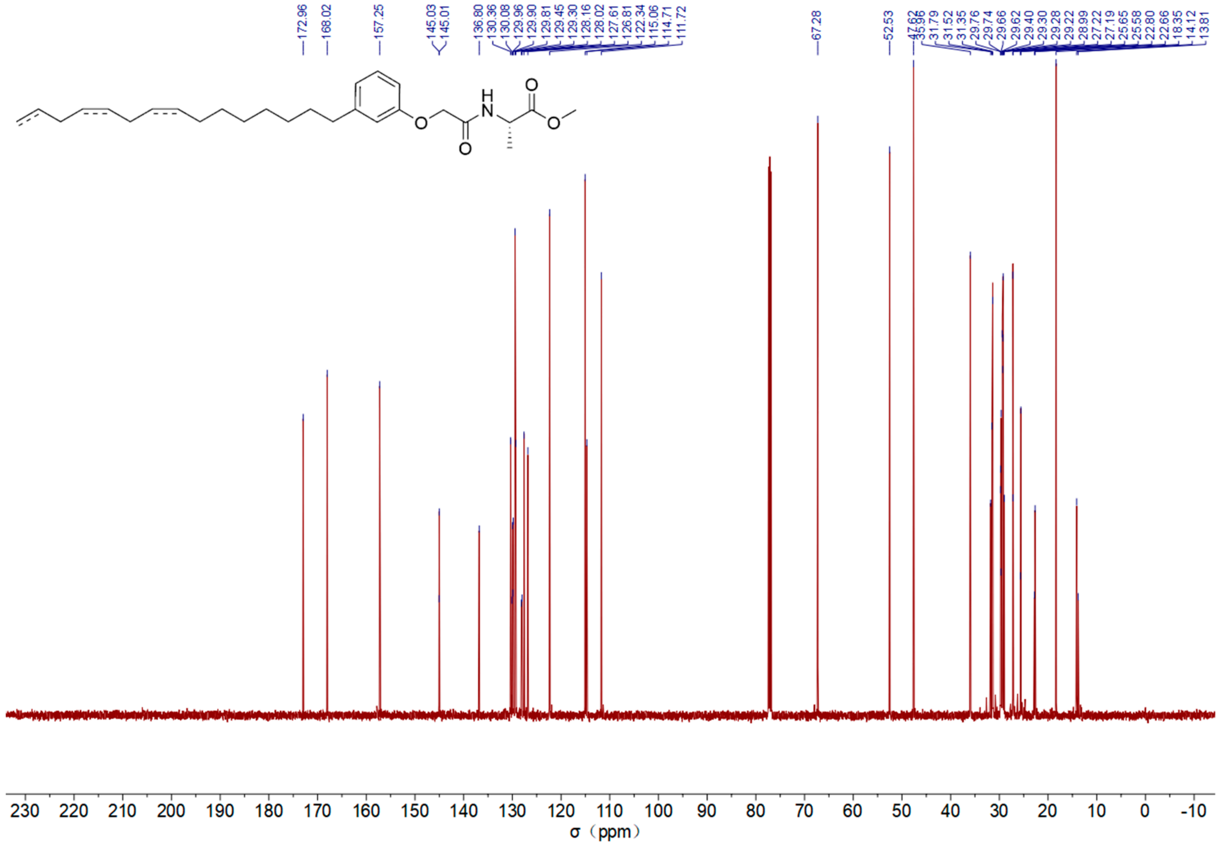

Figure S6 ^{13}C NMR spectra recorded (126 MHz, $CDCl_3$, r.t.) for **4a**

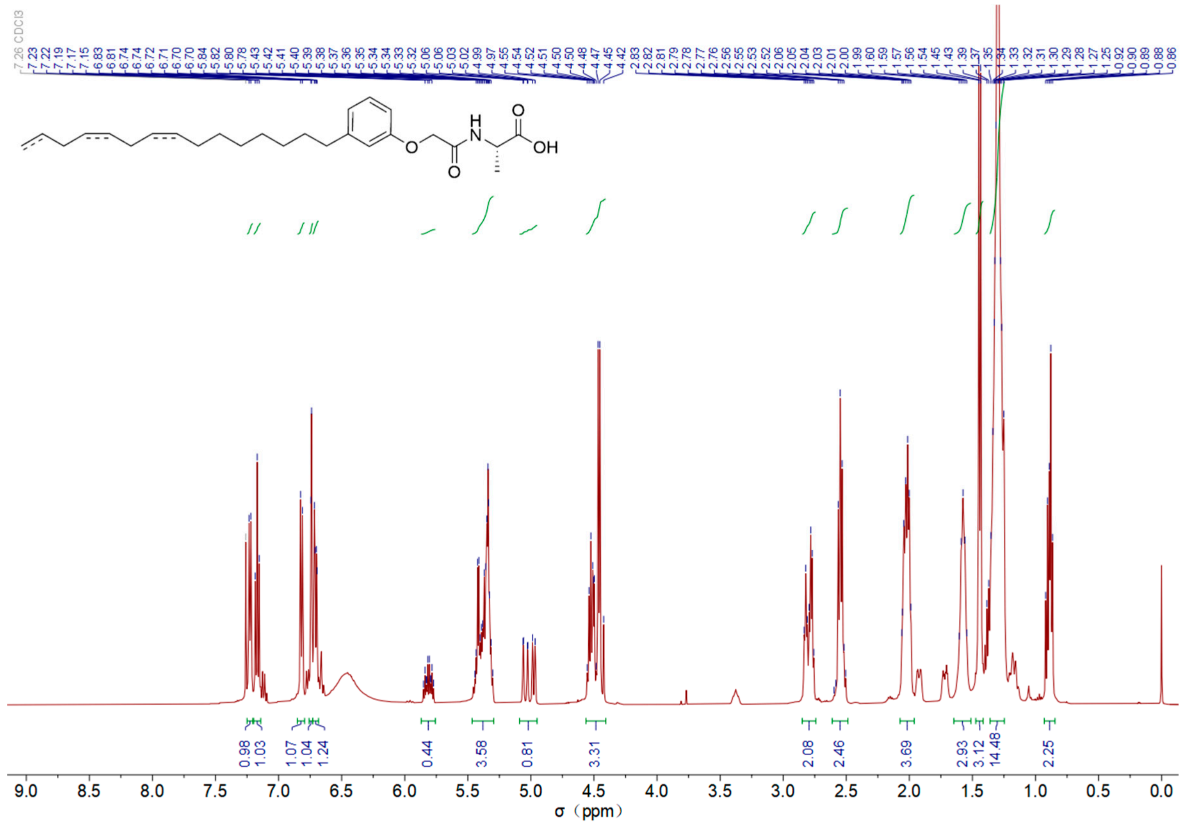

Figure S7 ^{1}H NMR spectra recorded (500 MHz, $CDCl_3$, r.t.) for **CALAH**

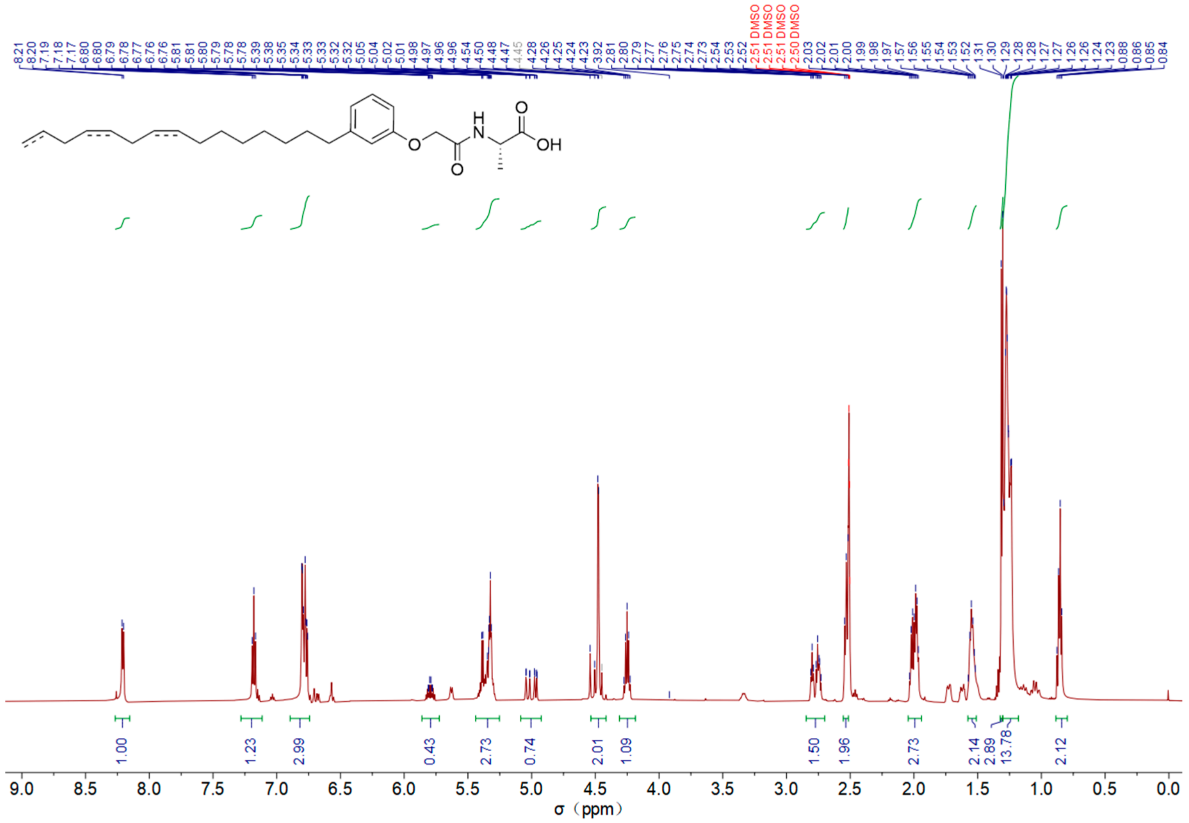

Figure S8 ^{1}H NMR spectra recorded (600 MHz, DMSO-d_6, r.t.) for **CALAH**

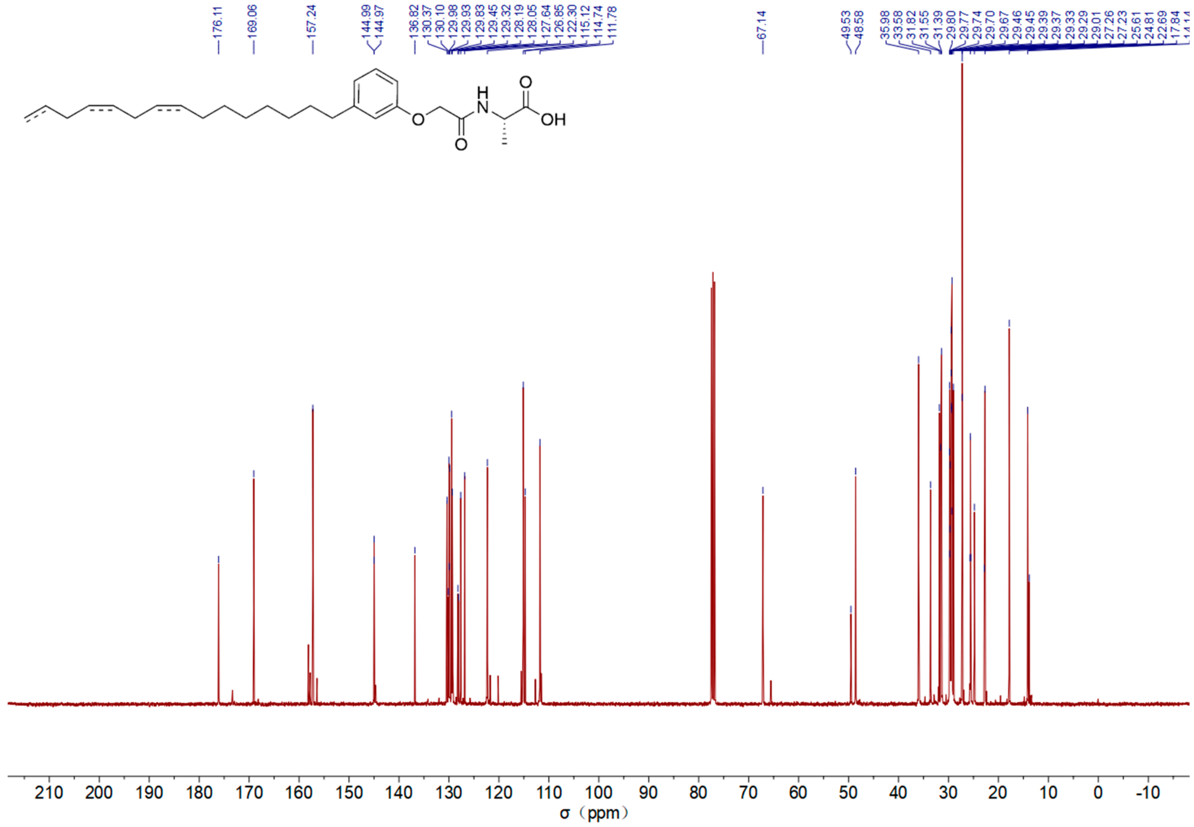

Figure S9 ^{13}C NMR spectra recorded (101 MHz, $CDCl_3$, r.t.) for **CALAH**

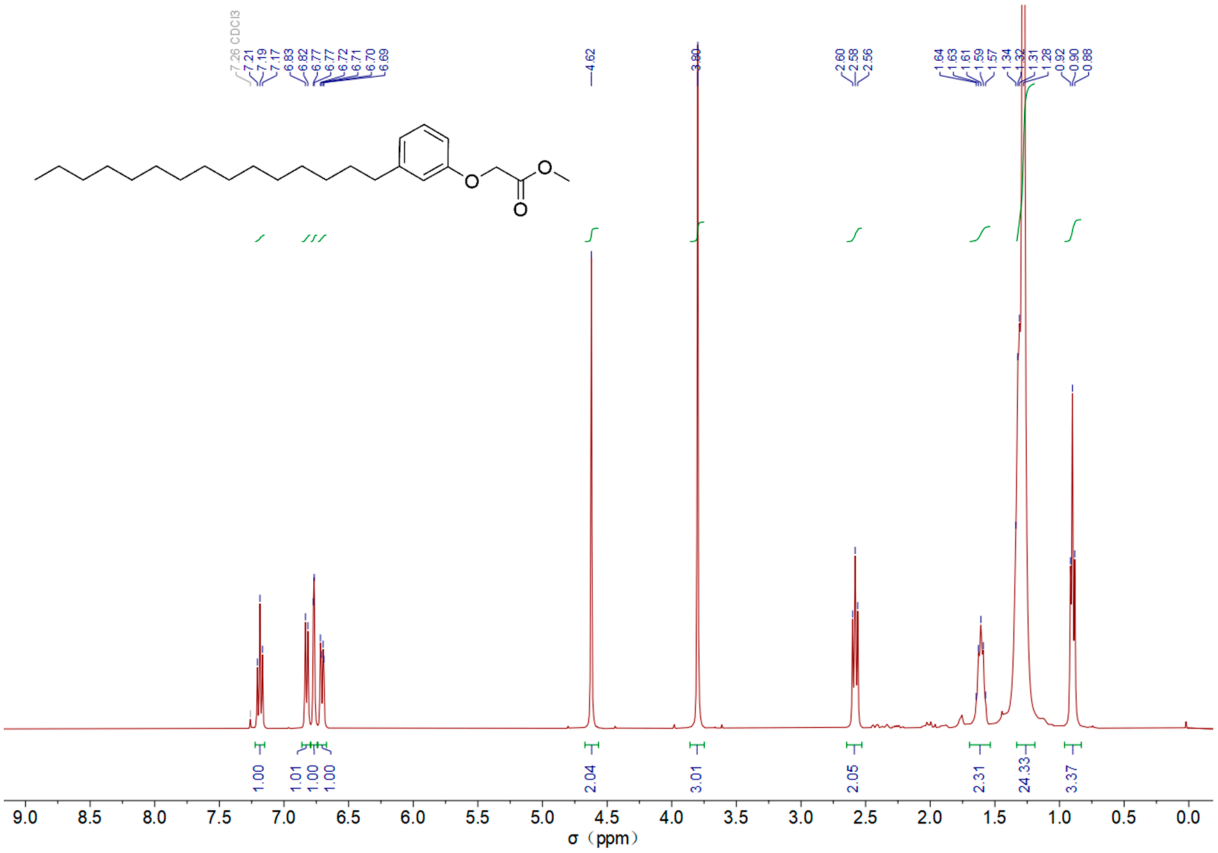

Figure S10 ^{1}H NMR spectra recorded (400 MHz, $CDCl_3$, r.t.) for **2b**

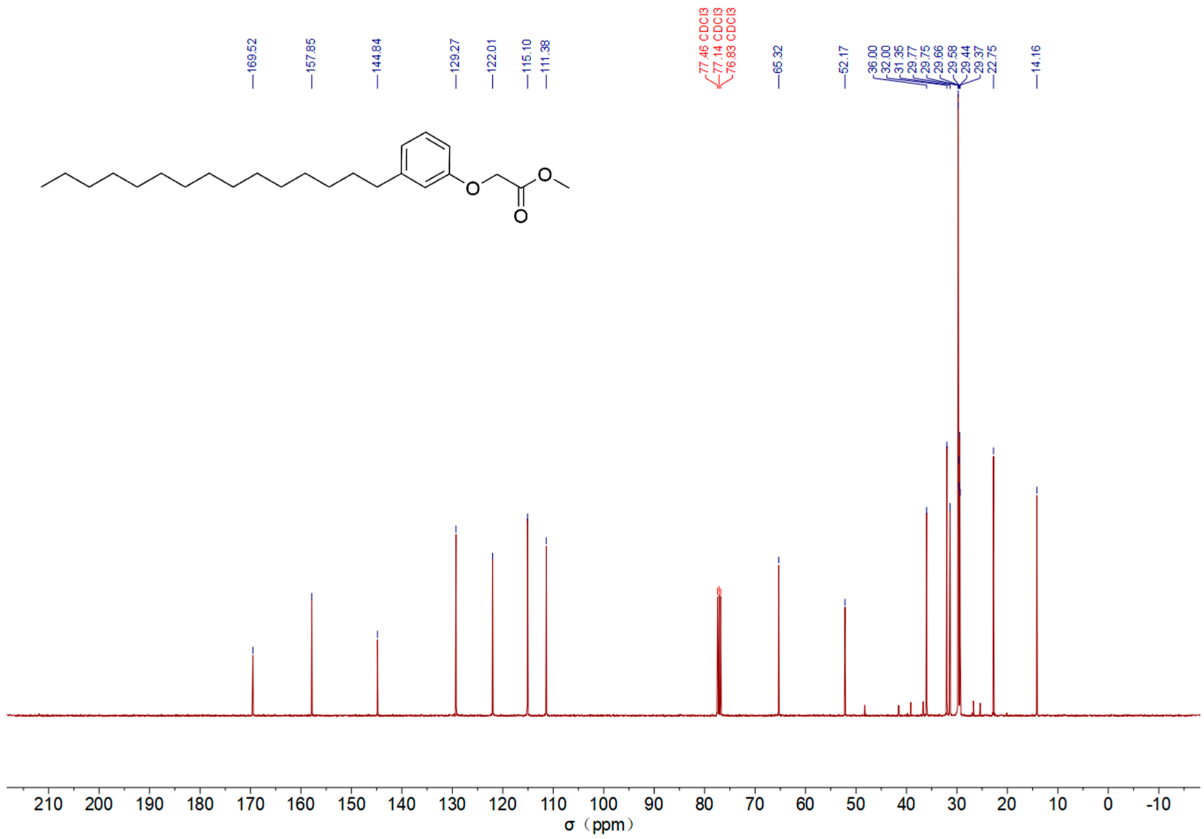

Figure S11 ^{13}C NMR spectra recorded (126 MHz, CDCl3, r.t.) for **2b**

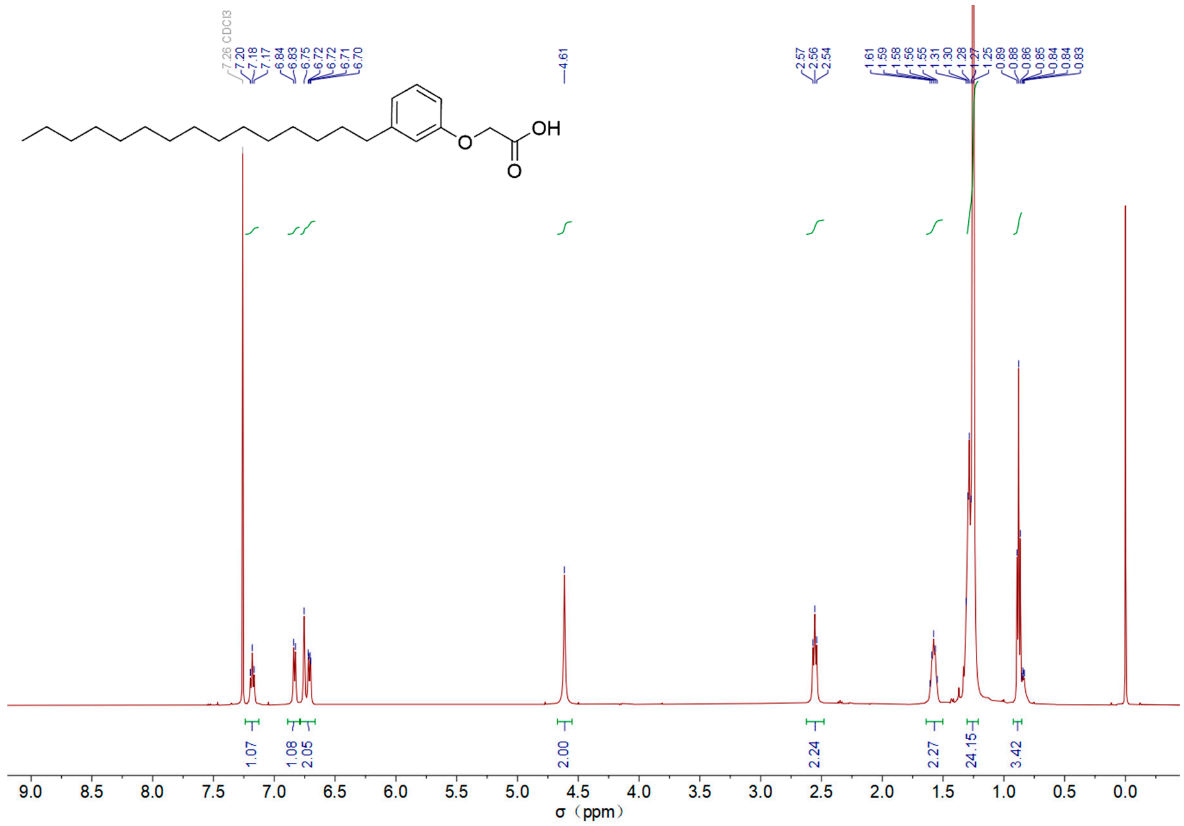

Figure S12 ^{1}H NMR spectra recorded (500 MHz, CDCl3, r.t.) for **3b**

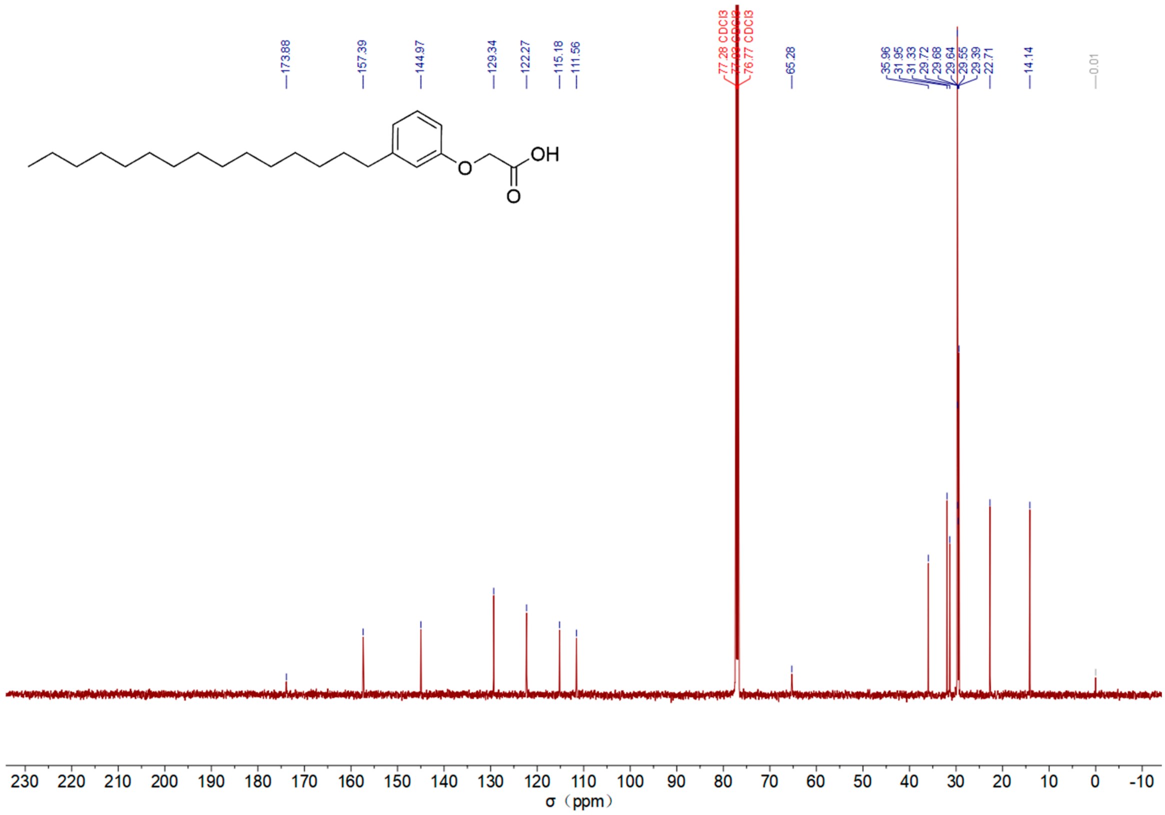

Figure S13 ^{13}C NMR spectra recorded (126 MHz, $CDCl_3$, r.t.) for **3b**

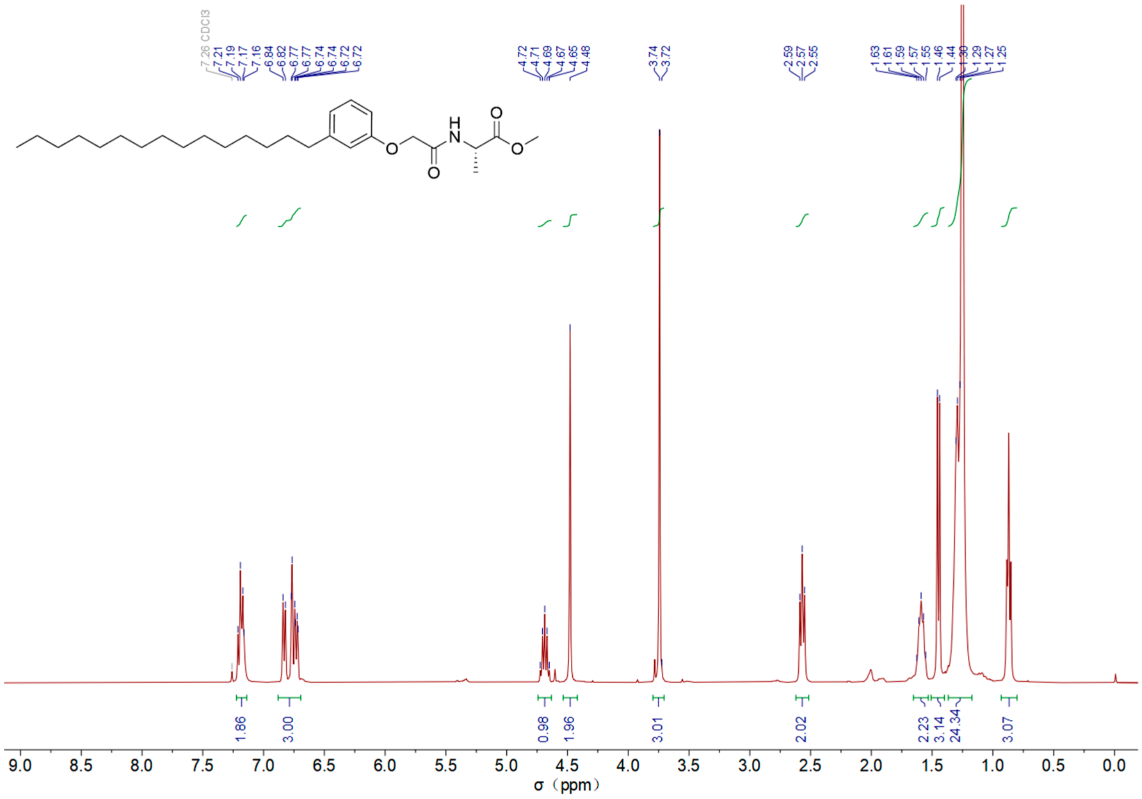

Figure S14 ^{1}H NMR spectra recorded (400 MHz, $CDCl_3$, r.t.) for **4b**

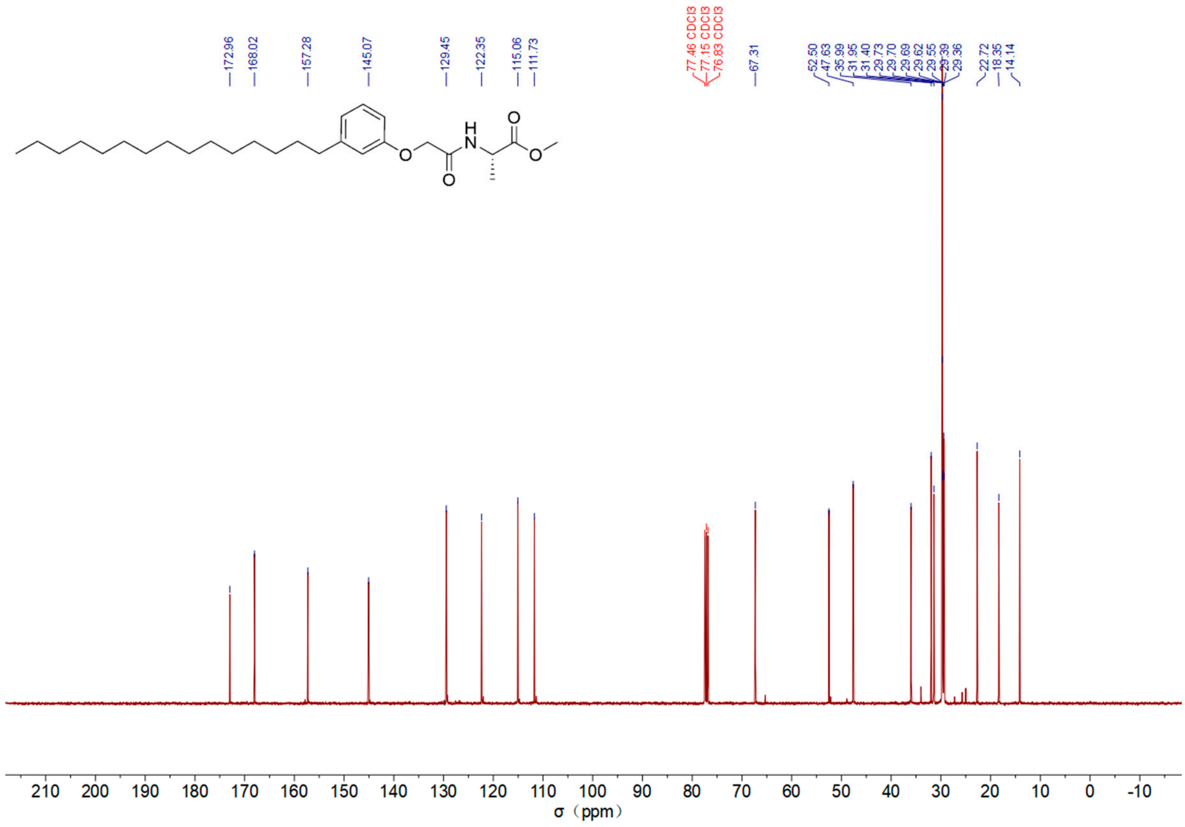

Figure S15 ^{13}C NMR spectra recorded (101 MHz, $CDCl_3$, r.t.) for **4b**

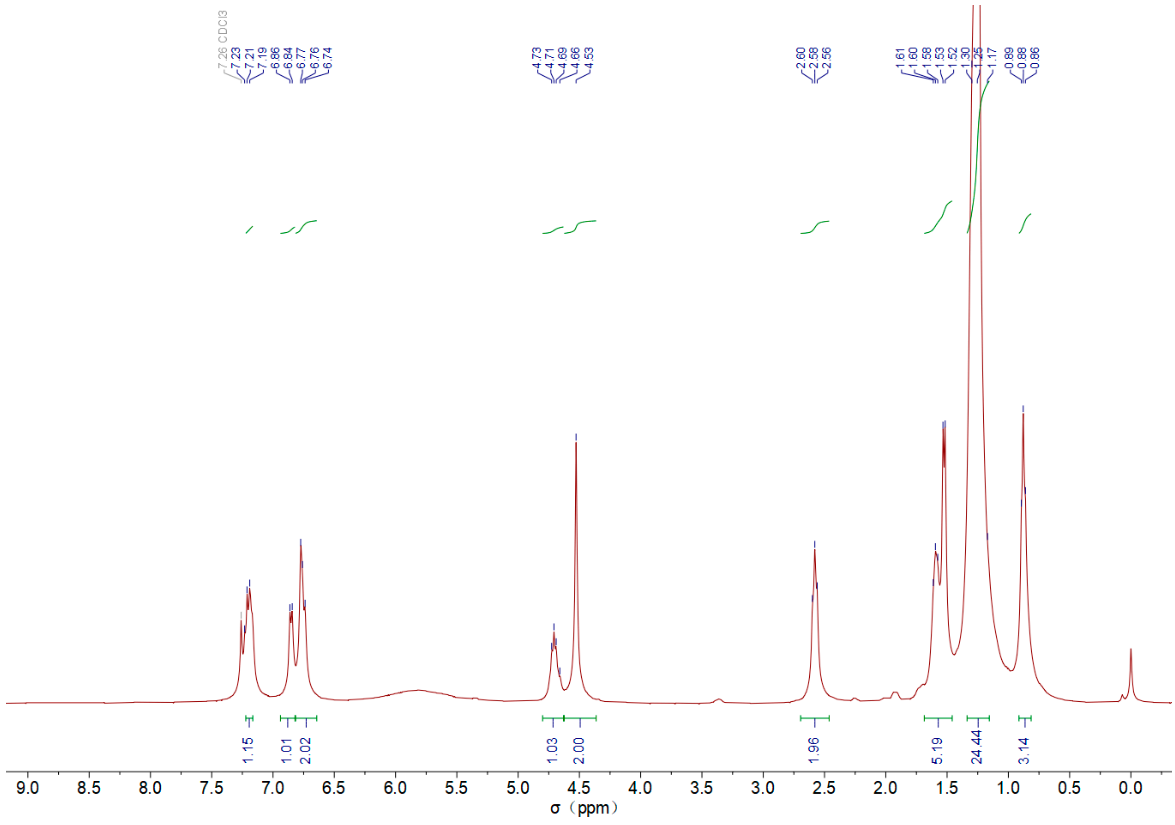

Figure S16 ^{1}H NMR spectra recorded (400 MHz, $CDCl_3$, r.t.) for **PALAH**

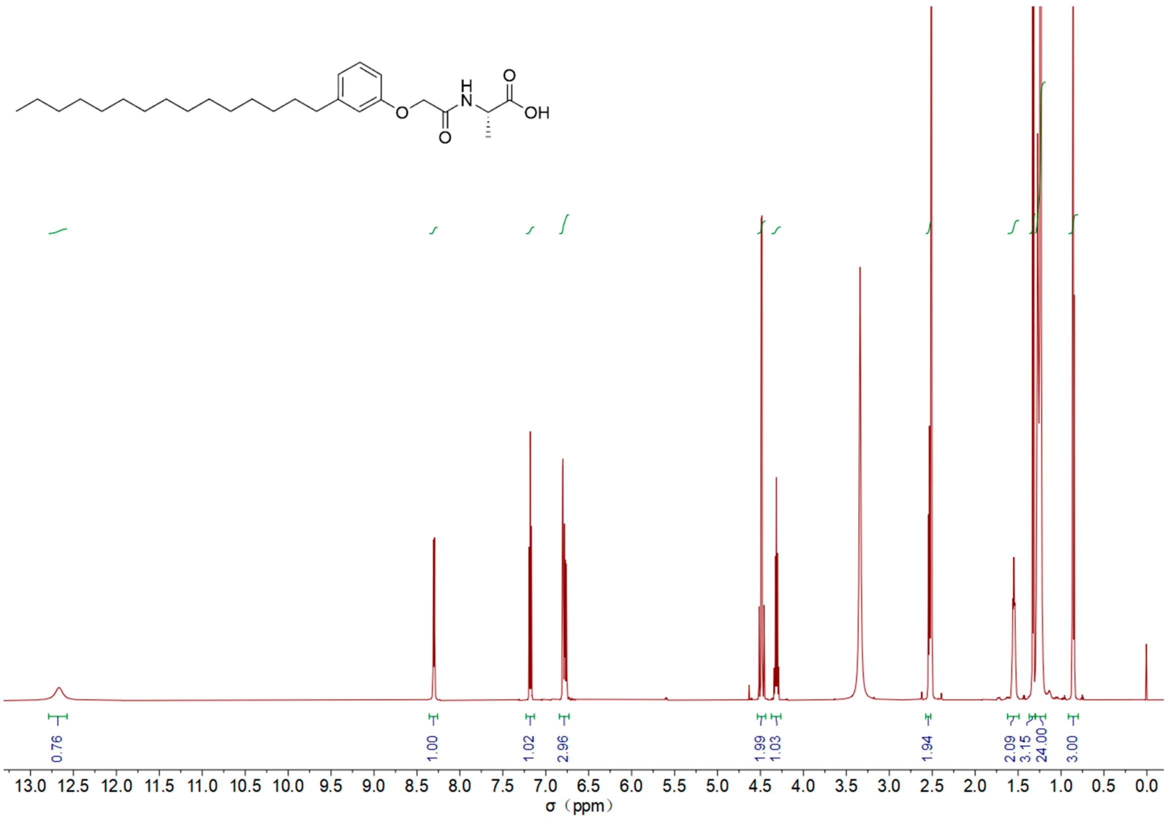

Figure S17 ^{1}H NMR spectra recorded (600 MHz, DMSO-*d*$_6$, r.t.) for **PALAH**

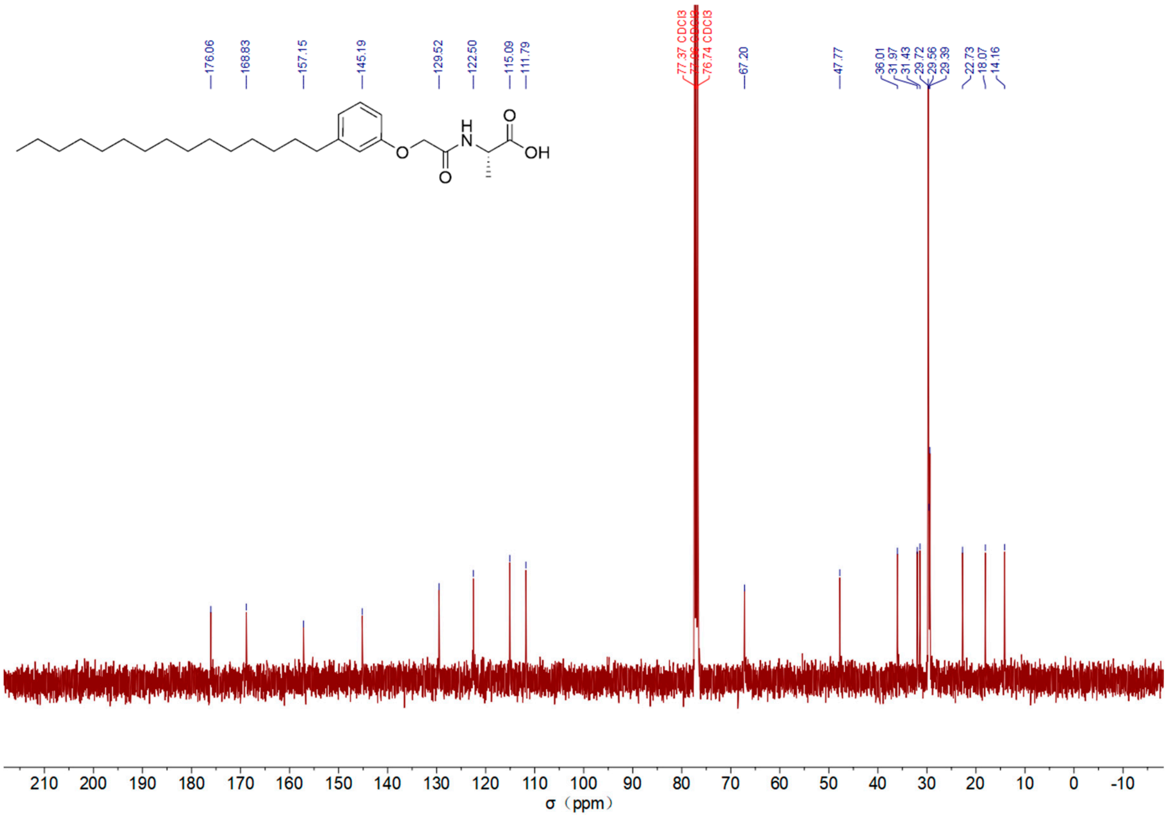

Figure S18 ^{13}C NMR spectra recorded (101 MHz, $CDCl_3$, r.t.) for **PALAH**

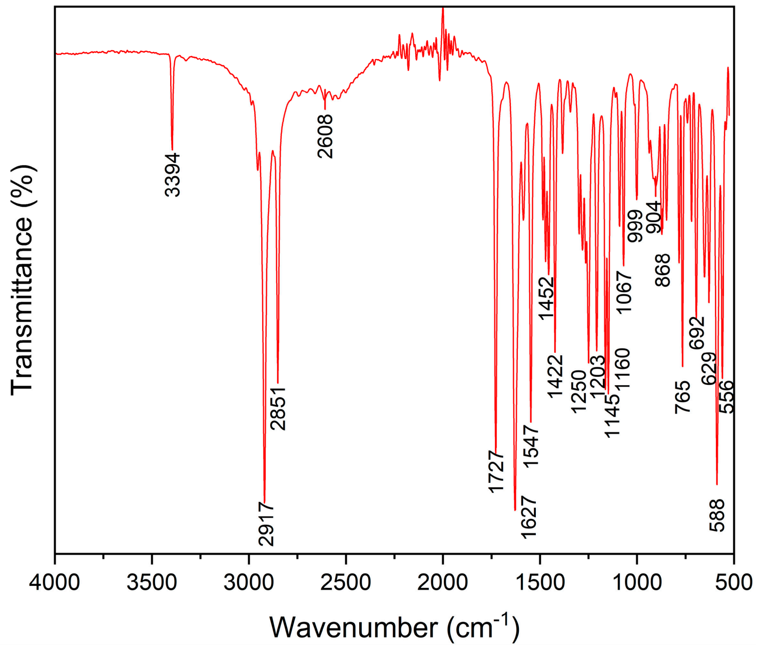

Figure S19 IR spectra recorded for **PALAH**

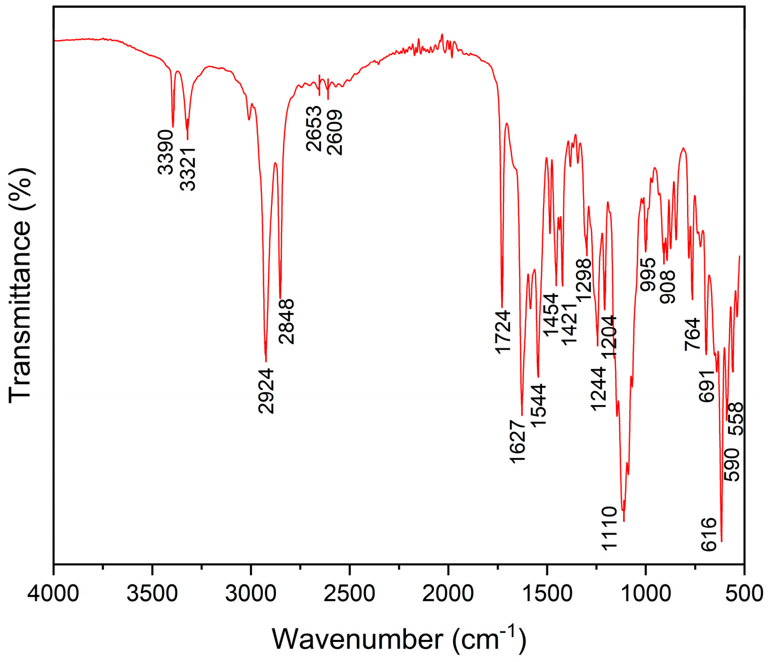

Figure S20 IR spectra recorded for **CALAH**

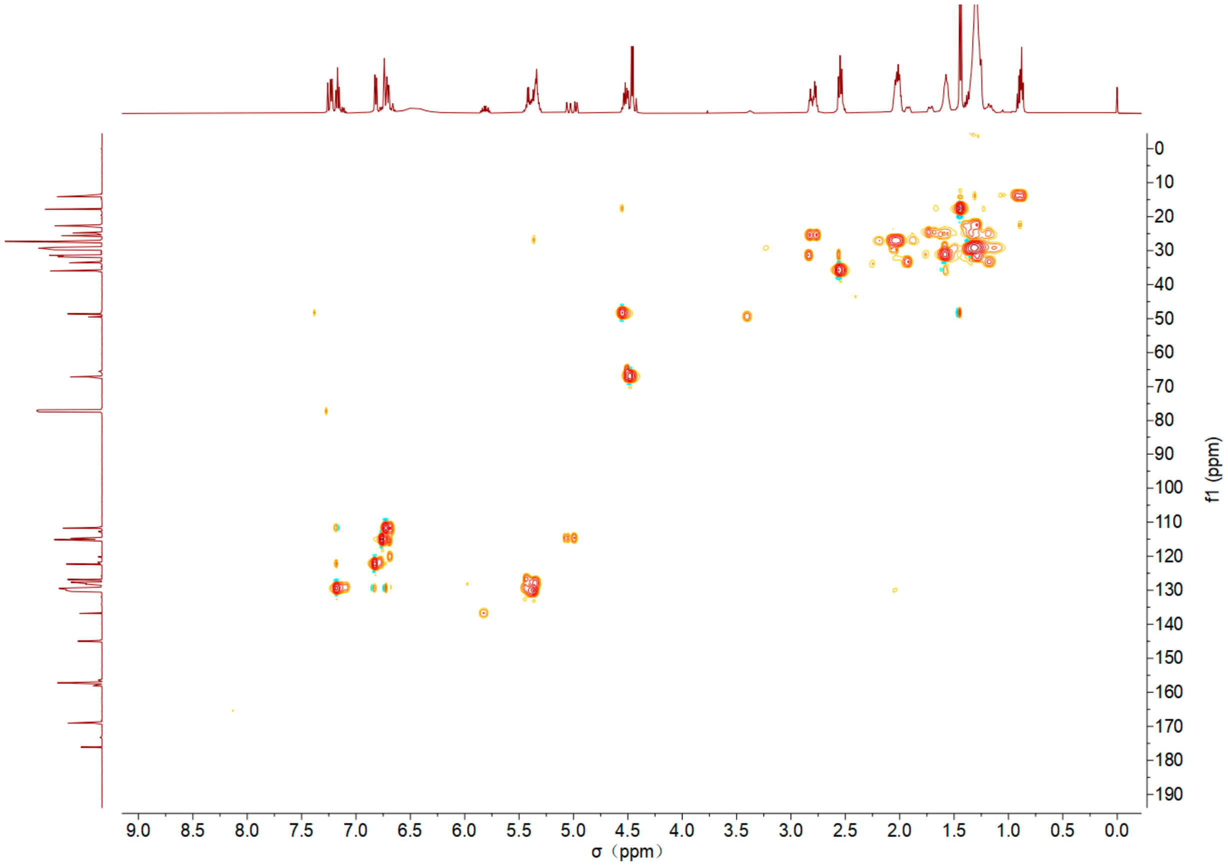

Figure S21 2D HSQC NMR spectra recorded (600 MHz, $CDCl_3$, r.t.) for **CALAH**

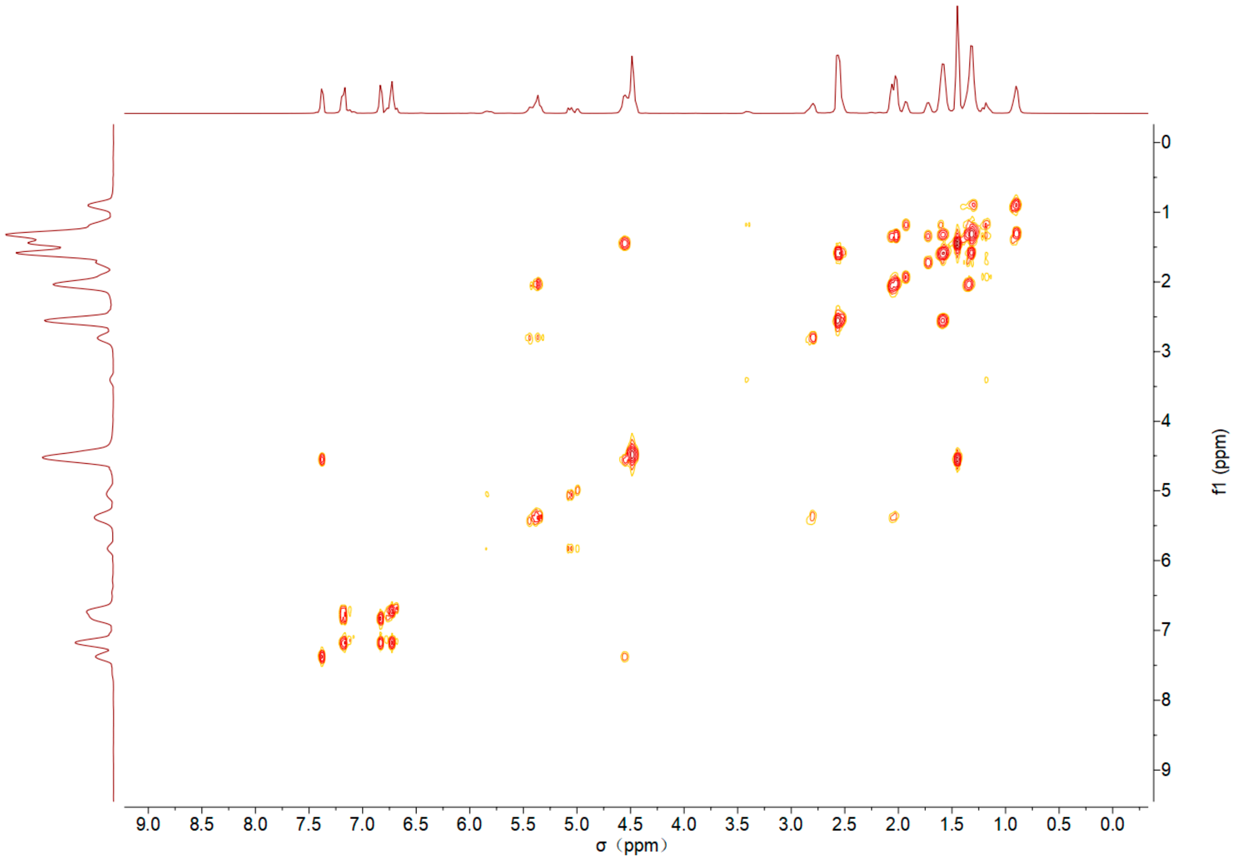

Figure S22 2D NOESY NMR spectra recorded (600 MHz, $CDCl_3$, r.t.) for **CALAH**

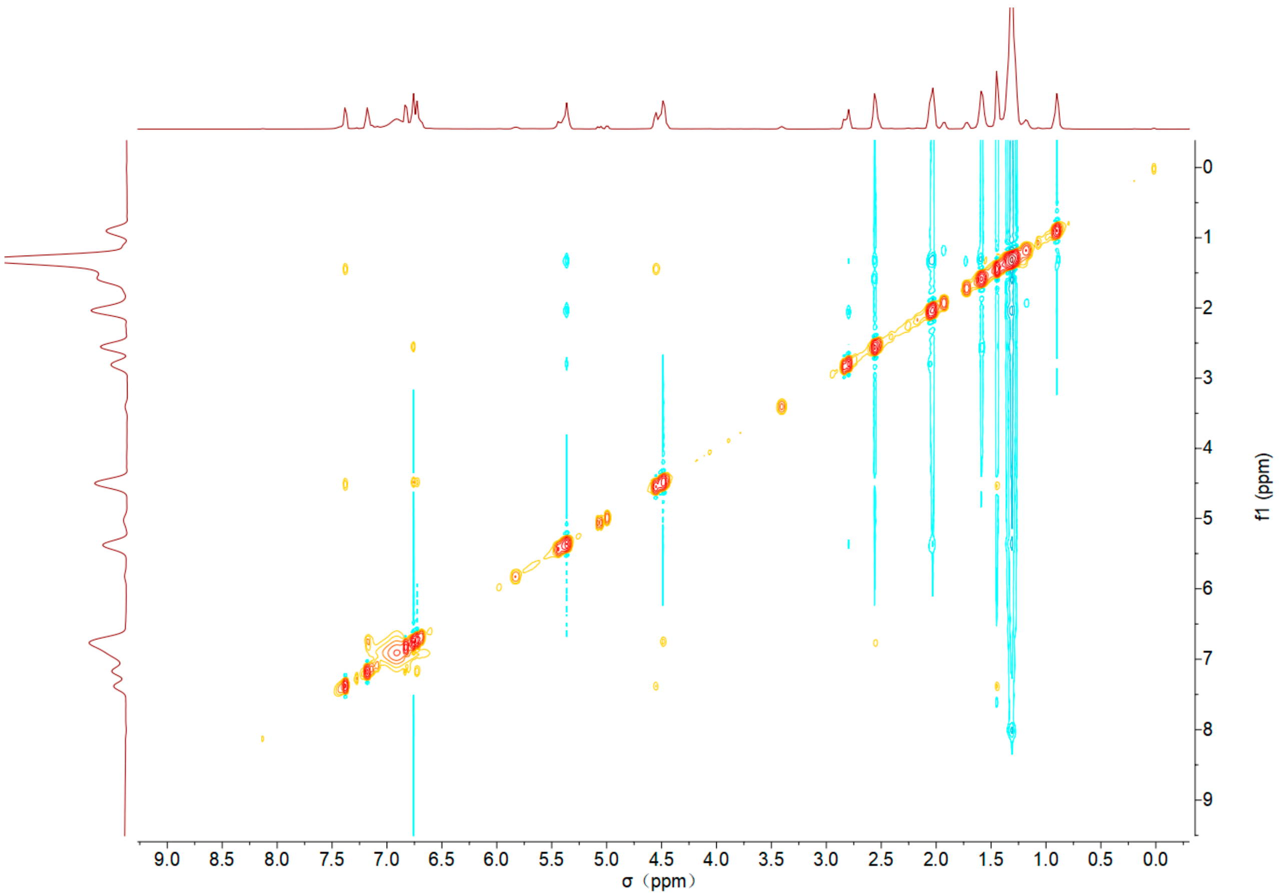

Figure S23 2D COSY NMR spectra recorded (600 MHz, $CDCl_3$, r.t.) for **CALAH**

Morphological structures

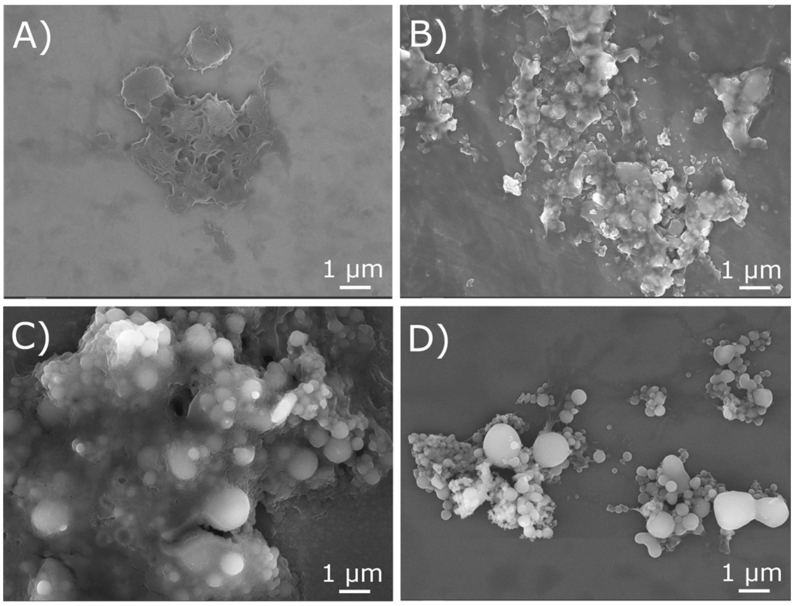

Figure S24 SEM images of **PALA**, (A, B) and **CALA** (C, D) respectively.

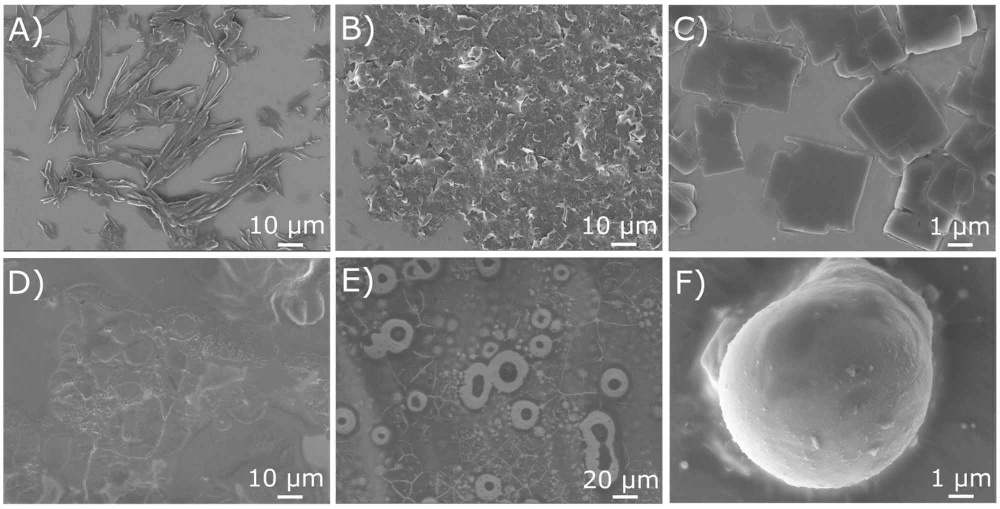

Figure S25 SEM images of **PALAH**, (A, B, C) and **CALAH** (D, E, F) in ethyl acetate, respectively.

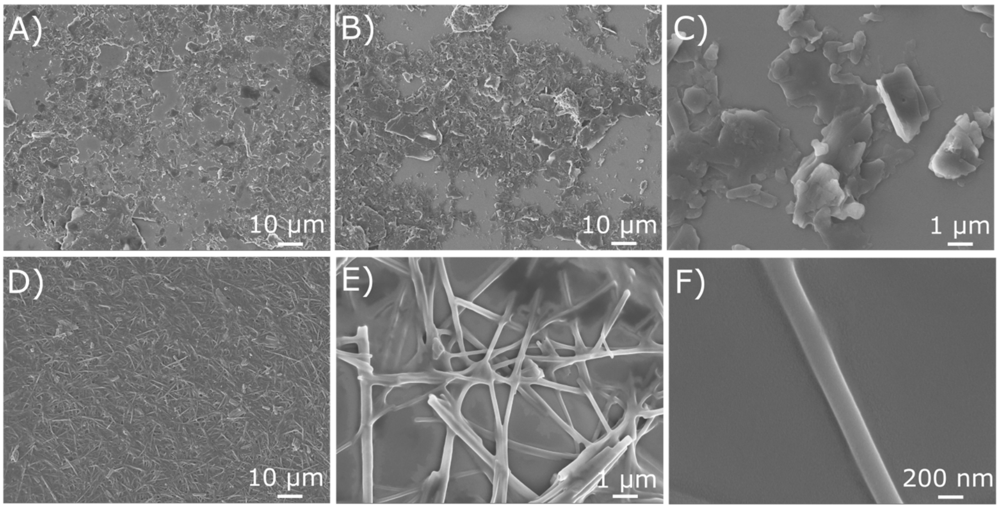

Figure S26 SEM images of **PALAH**, (A, B, C) and **CALAH** (D, E, F) in cyclohexane, respectively.

^{1}H NMR self-assembly analysis

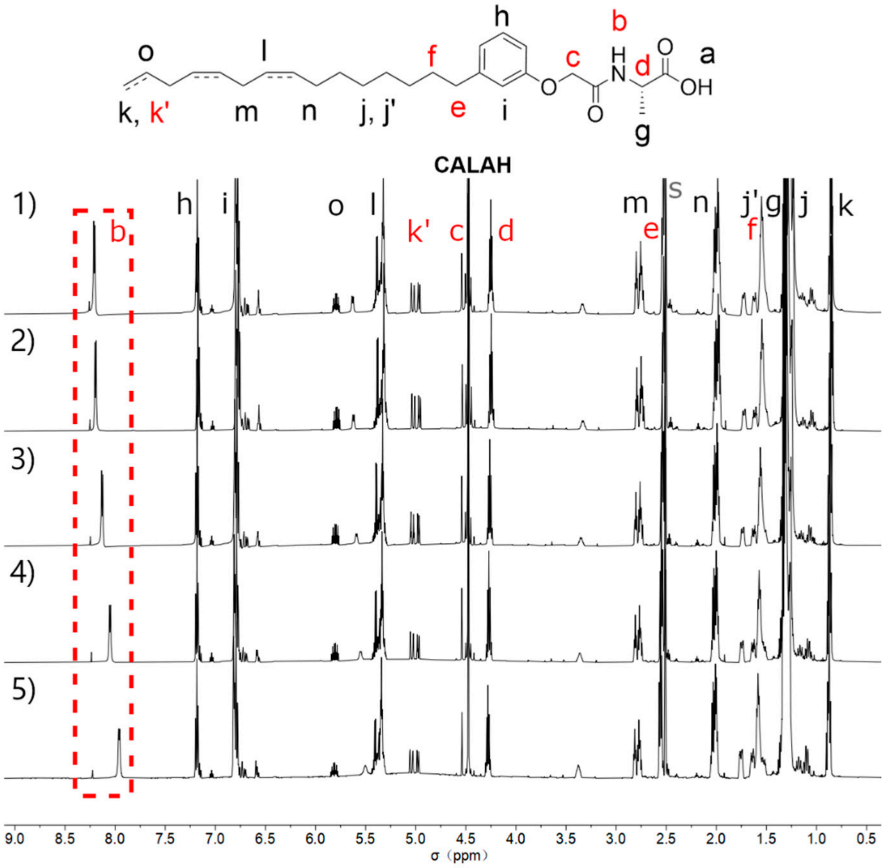

Figure S27 ^{1}H NMR spectra of temperature-dependent (600 MHz, DMSO-*d*6, ppm) for **CALAH**, 1) 10 °C, 2) 25 °C, 3) 40 °C, 4) 55 °C, 5) 70 °C.

^{1}H NMR self-assembly structures

Table S1 The specific rotation of **PALAH**/**CALAH**

Compounds	Concentration (g/100 mL)	Specific rotation [α]			
		1	2	3	Average
**PALAH**	0.025	293.662	294.987	291.537	293.395
**CALAH**	0.025	290.241	292.898	294.244	292.461

DFT calculation

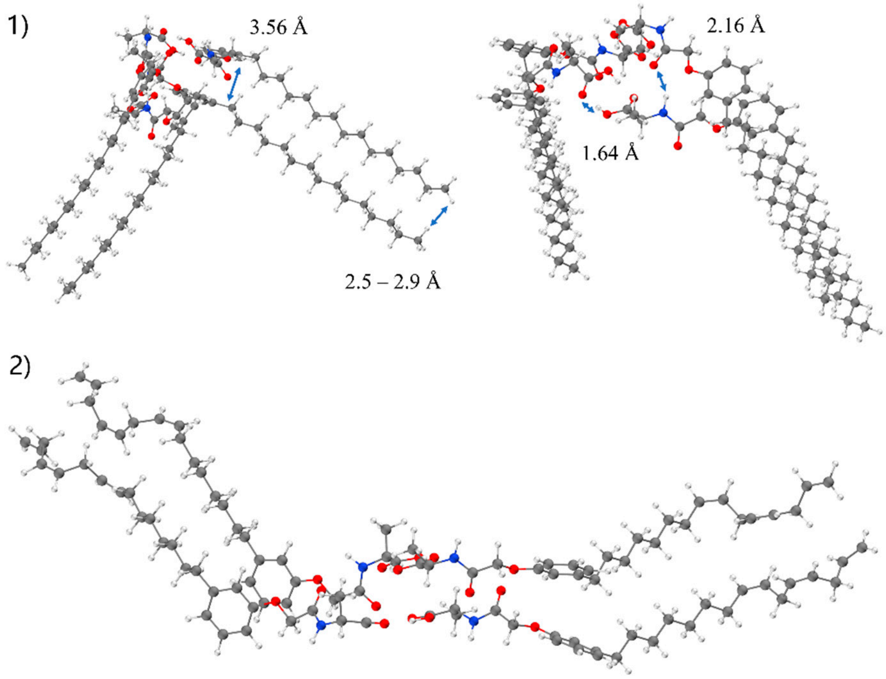

Figure S28 The DFT model of **PALAH** (1) and **CALAH** (2).

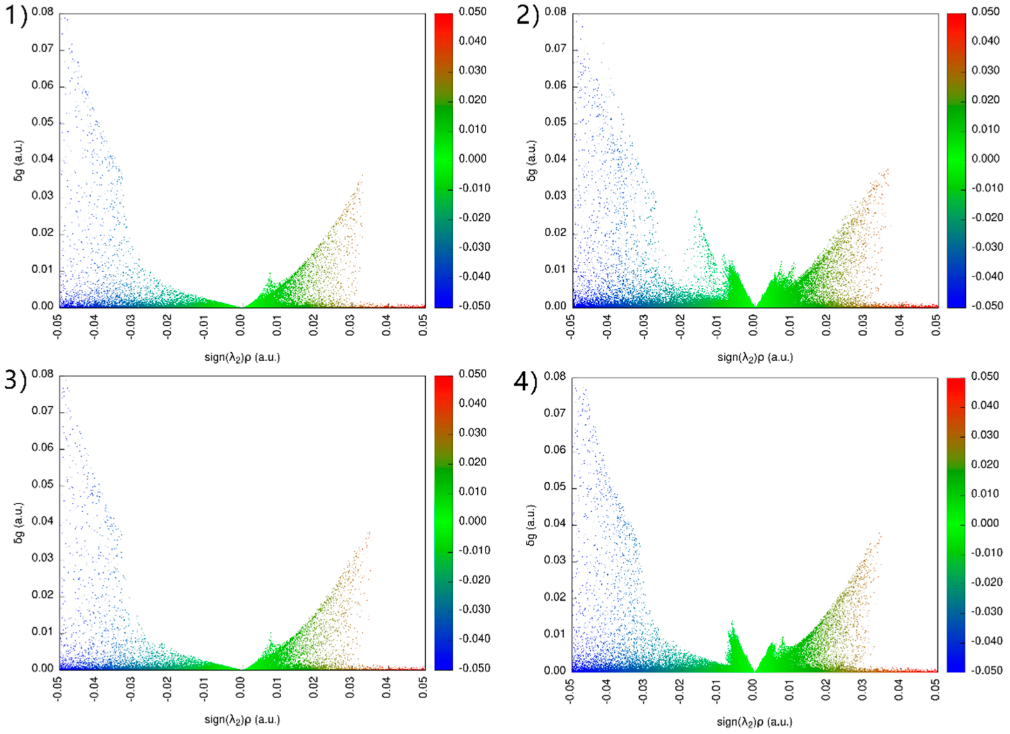

Figure S29 The scatter plot of RDG vs sign(lambda2)rho of dimer/tetramer for **PALAH**/**CALAH**.

Cell and microbial inhibitory properties

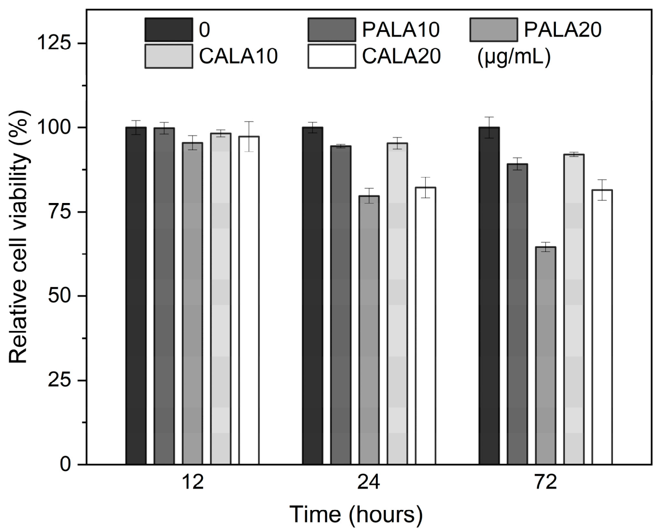

Figure S30 In vitro cytotoxicity of **PALA** and **CALA** against L929 cell line.
